# A multi-omics reciprocal analysis for characterization of bacterial metabolism

**DOI:** 10.3389/fmolb.2025.1515276

**Published:** 2025-03-20

**Authors:** Gabriel Santos Arini, Tiago Cabral Borelli, Elthon Góis Ferreira, Rafael de Felício, Paula Rezende-Teixeira, Matheus Pedrino, Franciene Rabiço, Guilherme Marcelino Viana de Siqueira, Luiz Gabriel Mencucini, Henrique Tsuji, Lucas Sousa Neves Andrade, Leandro Maza Garrido, Gabriel Padilla, Alberto Gil-de-la-Fuente, Mingxun Wang, Norberto Peporine Lopes, Daniela Barretto Barbosa Trivella, Letícia Veras Costa-Lotufo, María-Eugenia Guazzaroni, Ricardo Roberto da Silva

**Affiliations:** ^1^ Computational Chemical Biology Laboratory, Department of BioMolecular Sciences, School of Pharmaceutical Sciences of Ribeirão Preto, University of São Paulo, São Paulo, Brazil; ^2^ NPPNS, Department of BioMolecular Sciences, School of Pharmaceutical Sciences of Ribeirão Preto, University of São Paulo, São Paulo, Brazil; ^3^ Cellular and Molecular Biology Program, Department of Cellular and Molecular Biology of Ribeirão Preto, School of Medicine, University of São Paulo, São Paulo, Brazil; ^4^ Marine Pharmacology Laboratory, Department of Pharmacology, Institute of Biomedical Sciences, University of São Paulo, São Paulo, Brazil; ^5^ Brazilian Biosciences National Laboratory (LNBio), Brazilian Center for Research in Energy and Materials (CNPEM), Campinas, Brazil; ^6^ MetaGenLab Laboratory, Department of Biology, FFCLRP, University of São Paulo of Ribeirão Preto, School of Medicine, University of São Paulo, São Paulo, Brazil; ^7^ Laboratory of Bioproducts, Department of Microbiology, Institute of Biomedical Sciences, University of São Paulo, São Paulo, Brazil; ^8^ Centro de Metabolómica y Bioanálisis (CEMBIO), Facultad de Farmacia, Universidad San Pablo-CEU, CEU Universities, Urbanización Montepríncipe, Boadilla del Monte, Spain; ^9^ Departamento de Tecnologías de la Información, Escuela Politécnica Superior, Universidad San Pablo-CEU, CEU Universities, Urbanización Montepríncipe, Boadilla del Monte, Spain; ^10^ Department of Computer Science and Engineering, University of California Riverside, Riverside, CA, United States

**Keywords:** untargeted metabolomics, genomics, multi-omics analysis, bioinformactics, microbiology

## Abstract

**Introduction:**

Exploiting microbial natural products is a key pursuit of the bioactive compound discovery field. Recent advances in modern analytical techniques have increased the volume of microbial genomes and their encoded biosynthetic products measured by mass spectrometry-based metabolomics. However, connecting multi-omics data to uncover metabolic processes of interest is still challenging. This results in a large portion of genes and metabolites remaining unannotated. Further exacerbating the annotation challenge, databases and tools for annotation and omics integration are scattered, requiring complex computations to annotate and integrate omics datasets.

**Methods:**

Here we performed a two-way integrative analysis combining genomics and metabolomics data to describe a new approach to characterize the marine bacterial isolate BRA006 and to explore its biosynthetic gene cluster (BGC) content as well as the bioactive compounds detected by metabolomics.

**Results and Discussion:**

We described BRA006 genomic content and structure by comparing Illumina and Oxford Nanopore MinION sequencing approaches. Digital DNA:DNA hybridization (dDDH) taxonomically assigned BRA006 as a potential new species of the Micromonospora genus. Starting from LC-ESI(+)-HRMS/MS data, and mapping the annotated enzymes and metabolites belonging to the same pathways, our integrative analysis allowed us to correlate the compound Brevianamide F to a new BGC, previously assigned to other function.

## 1 Introduction

The search for new bioactive compounds of natural origin from different organisms coming from different biomes is an arduous task. Since marine environments are poorly explored, they hold the promise of a formidable source of rich metabolic potential for the production of novel biosynthetic compounds, especially when you consider the microorganisms that reach a billion strains in a Gram of marine sediment (Fenical and Jensen, 2006). Considering that more than 40% of pharmaceutical ingredients are derived directly or indirectly from natural products derived from plants or microorganisms, one can expect that thousands of unknown potential medicines are expected to be discovered in marine ecosystems (Newman and Cragg, 2020; Kim et al., 2021). This feature places Brazil under the spotlight since its coast is especially large, ranging from tropical to temperate climate zones (Wilke et al., 2021).

The Micromonospora genus is composed by 177 Gram-positive, spore-forming aerobic species found mainly in marine environments. Also, this genus belongs to the phylum Actinomycetota, which is responsible for 70% of natural compounds under development or already in clinical use (Hifnawy et al., 2020). The chemical diversity, in terms of natural products, that this genus is capable of producing is enormous. *Micromonospora* natural products are used as drugs against infections caused by fungi or bacteria. The genera started to receive attention after the discovery of gentamicin in 1963 and after that, more than 740 bioactive compounds have been reported from *Micromonospora* strains. Among this chemical diversity produced, as well as the different locations where this genus can be found, there are reports in the literature searching specifically for anticancer bioactive compounds on *Micromonospora sp*. BRA006 (Sousa et al., 2012).

The evolution of bacterial genome organization clustered genes that encode enzymes of the same metabolic pathway, which are known as biosynthetic gene clusters (BGC) (Ruzzini and Clardy, 2016). For this reason, modern drug discovery from bacteria is based on BGC identification as the starting point followed by an experimental procedure that aims to detect, isolate, or produce the compound (Domingues Vieira et al., 2022). However, searching for novel natural bioactive compounds from microorganisms can be a harsh task, mostly because the majority of the microbial life cannot be cultured under laboratory conditions. Also, it is difficult to obtain bioactive compounds in the desired concentrations (Sun et al., 2019). Thus, bacterial bioactive compound discovery requires a multidisciplinary approach, such as genomics and metabolomics (Behsaz et al., 2021). Genomics enables the analysis of the whole genome sequencing data and raises hypotheses about metabolic pathways and compound products based on the genetic content (Andrade, 2020). Then, metabolomic assays based on mass spectrometry analysis, such as LC-MS/MS are performed to validate them as well as be a important step in order to identify new possible bioactive compounds. The integration of these two omics sciences through multi-omics approaches opens up the possibility of accessing how much of a given compound predicted in a BGC is actually being produced and vice versa. And also to search for new BGCs starting from the annotated metabolites. Addtionaly, data integration has shown a promising resource in the description of new bacterial strains, as it allows the study and characterization of new microorganisms in a holistic way (Kato et al., 2024).

**FIGURE 1:**
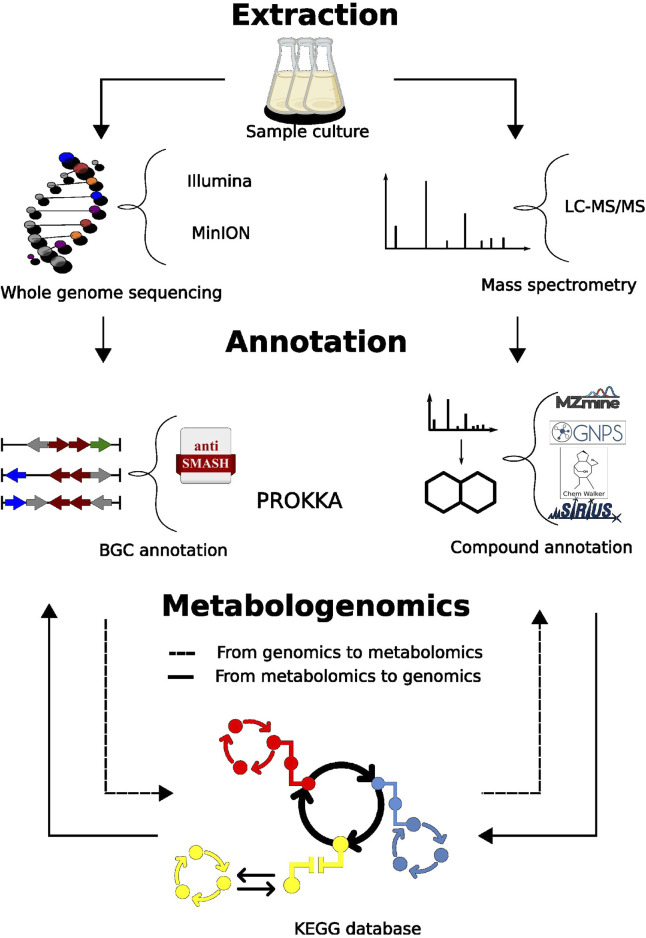
Metabologenomics workflow. LC-MS/MS raw data was pre-processed using MZmine3. Spectral pairing and molecular network construction with GNPS2. In silico annotations were performed with ChemWalker and SIRIUS. The annotated compounds were used to search KEGG pathway database. KEGG pathway matches had the enzymes serarched in the genome. The genome assemblies for MInION and Illumina had gene annotation performed by Prokka and AntiSMASH. From the prediction of metabolites, we searched the annotated metabolome.

In the present work, we used multi-omics approach to describe the potential bioactive compounds of BRA006 (Figure 1), a bacteria strain recovered from a marine environment collected on the coast of Brazil. We performed our analysis in a two-way direction, searching for metabolites by LC-MS/MS previously predicted by antiSMASH, using two genome sequencing platforms, as well as finding coding sequences (CDS) for enzymes that are part of metabolic pathways for syntheses of BRA006s observed metabolome.

## 2 Materials and methods

### 2.1 Collection, DNA extraction, and sequencing

The BRA006, from MicroMarin collection (https://www.labbmar.ufc.br/micromarinbr) was cultured in A1 medium [Starch (10 g/L); Yeast extract (4 g/L); Peptone (2 g/L); Sea Water 75%] in a volume of 100 mL in 250 mL Erlenmeyer flasks. The cultures were centrifuged at 12.000 × g for 10 min and the cell pellets were resuspended in 10 uL of lysozyme 0.05 g/mL for 500 ul of SET buffer. The mixture was incubated at 37°C for 30 min. Then 14 ul Proteinase K (20 mg/mL) and 60 uL SDS 10% were added to the cell lysate and incubated for a further 1 h at 55°C. Then 200 uL NaCl 5 M was added and the temperature was raised to 37°C. 500uL of chloroform was added and the system was centrifuged at 4,500 × g for 10min at 20°C. 500uL was collected in a new tube and 300uL of isopropanol was added and incubated overnight (16 h). The system was centrifuged at 14,000 × g for 10 min at 4°C. The supernatant was discarded and the DNA pellet was resuspended in TE buffer. DNA libraries were prepared using the Oxford Nanopore Ligation Sequencing Kit (SQK-LSK110) and library loading and sequencing were performed according to the manufacturer’s instructions and protocol for Flongle.

### 2.2 Genome sequencing and assembly pipeline

For MinION data in use an in-house pipeline with tools suggested by Oxford Nanopore. The acquisition of the raw data, a series of processing steps were followed until these genomes were assembled. The first stage of data processing consisted of converting the raw signals into DNA base sequences (base calling) using the Guppy tool (Wick et al., 2019) and Dorado with super high accuracy settings. With this data in hand, the quality of the readings obtained in the previous step was assessed using the NanoStat software (De Coster et al., 2018). With these results, the processing continued with the removal of adapters from the base called reads to prepare them for subsequent steps; this was done using the Porechop tool (https://github.com/rrwick/Porechop), which also compressed the resulting reads. Once the adapters had been removed, a new quality analysis was carried out using the NanoStat tool. Once this step was completed, the low-quality bases and short reads were trimmed using the Chopper tool (De Coster and Rademakers, 2023), which not only trimmed but also compressed the resulting reads. The next step was to analyze the quality of the resulting sequence, again using the NanoStat tool. With the resulting data, we then used the Flye (Kolmogorov et al., 2019) and Unycicler (Wick et al., 2017a) tools to assemble the genomes using filtered, high-quality reads. The genome assembly was then refined using information from reads mapped using the Racon tool (Vaser et al., 2017). Finally, with the final result in hand, the quality of the final genome assembly was assessed using the Quast tool (Gurevich et al., 2013) and BUSCO (Manni et al., 2021). For Illumina procedures, BRA006 genome samples were sequenced using MiSeq technology at Macrogen facility (Seul, South Korea), all the processing steps such as read mapping, trimming low quality reads and *de novo* genome assembly were performed using the proprietary software Geneious (Version 11) (Kearse et al., 2012).

### 2.3 Phylogenetic analysis

For phylogenetic inference, we choose a comprehensive method called digital DNA:DNA hybridization (dDDH) available in the Type Genome Server (TYGS) web tool (Meier-Kolthoff and Göker, 2019). We used both MinION and Illumina data as query sequences with a standard parameter set.

### 2.4 BGCs analysis and functional annotation

To reveal biosynthetic gene clusters (BGC) from the BRA006 genome, we used the antiSMASH tool (version 7.1.0) (Blin et al., 2023) with relaxed detection strictness and all extra features selection. Coding sequences (CDS) prediction of BRA006 assemblies were made using Prokka (1.14.6) (Seemann, 2014), which is based on prodigal (Hyatt et al., 2010) HMM models to identify proteins by their family motifs. Finally, we combine Prokka and antiSMASH results to obtain a better resolution of protein functions within BGCs. We manually filtered antiSMASH “Most Similar Known Cluster” feature and retrieved BGCs that matched MiBiG clusters related to functions of interest (version 3.0) (Terlouw et al., 2023). Thus, python scripts were used to convert CDS of GenBank files from MiBiG and antiSMASH into fasta format in which the MiBiG sequences were used to construct reference databases and antiSMASH’s as query sequences for BLASTp. With the TSV files from BLASTp, we grouped all possible matches by each query sequence and selected the one with the lowest e-value. Finally, we used BioPython to plot the comparisons with more than 70% of similarity.

### 2.5 Metabolomics analysis

The BRA006 isolates were cultivated in 100 mL of the sterile A1 culture medium (Starch 10 g/L, Yeast extract 4 g/L, Peptone 2 g/L and Sea Water 75%), in 250 mL Erlenmeyer flasks. After 5–7 days under 120 rpm agitation and 28°C, the liquid cultures were extracted with ethyl acetate (1:1) under agitation for 2 h, and the organic phase was dried under pressure and kept at 4°C. For the LC-MS/MS analyses, the organic extracts were diluted in methanol, and the extract solution to be injected was prepared in methanol:water solution at a ratio of 1:1 (v/v) with a final concentration of 1.0 mg/mL (Bauermeister et al., 2016)⁠. The LC-MS/MS analysis itself was conducted in the Acquity UPLC H-Class (Waters, Milford, MA - US) hyphenated with Impact II mass spectrometer (Bruker Daltonics, Billerica - US). The mobile phase (flow 0.3 mL.min-1) consisted of water (A) and methanol (B) in the following gradient: 0.0–15.0 min (5%–20% B, curve 6); 15.0–30.0 min (20%–95% B, curve 6); 30.0–33.0 min - (100 B, curve 1); 33.0–40.0 min (5% B, curve 1). C18 - Luna (Phenomonex® - 100 mm × 2.1 mm × 2.6 μm) and the temperature adjusted to 35°C. The parameters adjusted for the spectrometer were: end plate offset of 500V; capillary voltage of 4.5kV; nitrogen (N_2_) was used as gas; drying gas flow at 5.0 L.min^−1^; drying gas temperature at 180°C; 4 bar nebulizer gas pressure; positive ESI mode. Spectra (*m/z* 30–2000) were recorded at a rate of 8 Hz. The quadrupole ion energy was set to 5.0 eV. The collision cell was set to 5.0 eV, with collision energies ranging from 20 to 50 eV (transfer time from 20 to 70 μs), and absolute fragmentation cutoff of 1,000 counts. Ions below 200 Da were excluded from fragmentation. The “active exclusion” function was enabled and with the following settings: exclude after 3 spectra; release after 0.3 min; reconsider precursor if the ratio current intensity to previous intensity was 1.8 (Bazzano et al., 2024). Accurate masses were obtained using sodium formate solution (10 mM) as a calibrant (Hoang et al., 2024).

### 2.6 GNPS2 molecular networking and *in silico* annotation

The .csv and .mgf files generated in MZmine3 (Schmid et al., 2023) from the raw data of the metabolomic analysis were imported into the GNPS2 platform (https://gnps2.org/), where the molecular network and spectral pairing were performed using the Feature Based Molecular Network (FBMN) (Nothias et al., 2020) (https://gnps2.org/workflowinput?workflowname=feature_based_molecular_networking_workflow). We used the standard parameters for FBMN. Once the network was built, the annotations were propagated using the ChemWalker (Borelli et al., 2023) tool through GNPS2 interface (https://gnps2.org/workflowinput?workflowname=chemwalker_nextflow_workflow). We used the standard parameters for ChemWalker, including COCONUT (Sorokina et al., 2021) as the reference database and 0 for the component index to propagate information for the whole network. For the nodes that could not be annotated from these two previous methods, the MS/MS mass spectra of those compounds were analyzed using the SIRIUS tool version 5.8.5. To use this tool, we followed the annotation recommendations for Q-TOF mass analyzers and adopt the following adducts [M + H]^+^, [M + K]^+^, and [M + Na]^+^ as well as all the formulas available through the tool access (Dührkop et al., 2019). For *in silico* annotation, both ChemWalker and SIRIUS were used to annotate a structure with the best ranked candidate.

## 3 Results

### 3.1 Genomic analysis

The isolate BRA006 exhibits very characteristic growth. The colonies are orange in color with apical growth and a rough appearance with the presence of individual dark-colored spores. In solid medium, it releases as yet undetermined compounds that diffuse into the agar, giving it a pink-purple color, as can be seen in Supplementary Figure 1.

We compared Illumina and MinION whole genome sequence techniques results from BRA006. While Illumina assembly yielded 10 contigs and 6,734,372 base pairs (6.73 Mb) in total, sequencing by MinION showed higher genome completeness, with an assembly resulting in 4 contigs with 6,762,267 base pairs (6.76 Mb) in total. Functional annotation performed by Prokka showed a significant increase in CDS predicted from MinION assembly data driven by a high amount of hypothetical proteins. We corrected this issue adding modiciations in MinION assembly pipeline and obainted similar values to Illumina. The complete result is shown in (Table 1). The CDS prediction followed the number of hypothetical proteins, with more predictions from MinION than from Illumina.

**TABLE 1:** Summary information of Prokka and Busco tools applied on BRA006 genome.

Annotated proteins	With COG^a^	Hypothetical proteins	tRNA	rRNA	ncRNA	BUSCO^b^			Technique
						C	F	M	
11072	1,529	8,318	65	6	1	201	93	62	MinION
6,080	1769	3,289	69	5	1	350	1	5	Illumina
6,474	1807	3,601	68	9	1	328	17	11	MinION - corrected

This table contains the summary information of Prokka and Busco tools from the assembly of Illumina and MiniON, data sequencing techniques. The name “MinION, corrected” refers to the results of a troubleshooting process by Dorado and Unicycler, respectivly. To remove base calling errors from the original MiniION, sequencing data.

^a^ Complete distributions of COG (Cluster of Orthologous Groups) functional categories for each assembly technique.

^b^ Assembly completeness analysis based on near-universal single-copy orthologs gene content by BUSCO. The letters C, F, and M stand for complete, fragmented, and missing sequences, respectively.

### 3.2 Metabolic potential

*Micromonospora* is one of the largest genera of Actinomycetota and possesses a large repertoire of bioactive secondary metabolites (SM) with a broad spectrum of therapeutic effects (Yan et al., 2022), for instance, aminoglycosides and macrolactam antibiotics. Through antiSMASH, we annotated the BGC content from Illumina and MinION assemblies of BRA006. MinION assembly resulted in a total of 15 BGCs (Table 2) that vary in similarity with antiSMASH database. Among them, there are those with reported antimicrobial, antifungal, and antitumor activities. For instance, quinolidomicin A is a macrolide with antibiotic and anticancer effects isolated from *Micromonospora sp* JY16 (Ruzzini and Clardy, 2016) and BRA006 presents a quinolidomicin BGC with 219 Mb in length and 67% similarity with the most similar known cluster.

**TABLE 2:** Biosynthetic Gene Cluster from antiSMASH.

Region	Type	From	To	Most similar known cluster	Type from most simiilar known cluster	Similarity	Technique
1.1	T1PKS	173,653	262,468	catenulisporolides	NRP + Polyketide	12%	Illumina
1.2	T3PKS	972,718	1,013,770	loseolamycin A1/loseolamycin A2	Polyketide	92%/88%^a^	Both
1.3	thioamide-NRP	1,165,443	1,214,791	cadaside A/cadaside B	NRP	19%	Illumina
1.4	terpene	1,632,755	1,652,207	isorenieratene	Terpene	25%/25%^a^	Both
1.5	terpene	1,735,876	1,756,185	phosphonoglycans	Saccharide	3%	Illumina
1.9	T1PKS	3,818,978	3,889,418	quinolidomicin A	Polyketide	45%/67%^a^	Both
1.10	NI-siderophore	3,941,102	3,951,331	FW0622	Other	50%	Illumina
1.11	NRPS-like,NRPS,T1PKS,PKS-like	3,956,633	4,058,605	sungeidine C/sungeidine B/sungeidine D/sungeidine H/sungeidine A/sungeidine E/sungeidine F/sungeidine G	Polyketide	100%	Illumina
1.12	NRPS-like,NRPS,T1PKS	4,153,951	4,219,351	crochelin A	NRP + Polyketide	12%	Illumina
1.13	terpene	4,832,538	4,853,269	nocathiacin	RiPP:Thiopeptide	4%/4%^a^	Both
1.14	T2PKS	5,027,025	5,098,322	formicamycins A-M	Polyketide	18%	Illumina
1.15	oligosaccharide, terpene	5,268,386	5,304,696	lobosamide A/lobosamide B/lobosamide C	Polyketide	13%	Illumina
1.16	T2PKS,oligosaccharide, other,NRPS	5,309,290	5,436,713	cinerubin B	Polyketide:Type II polyketide	80%/74%	Both
1.17	NI-siderophore	5,616,661	5,629,872	peucechelin	NRP	10%	Illumina
1.18	terpene,RiPP-like	5,837,876	5,861,979	lymphostin/neolymphostinol B/lymphostinol/neolymphostin B	NRP + Polyketide	33%	Illumina
1.19	terpene	5,994,482	6,015,432	tetrachlorizine	Polyketide	13%	Illumina
1.20	other, ladderane,NRPS, arylpolyene	6,201,575	6,311,984	kedarcidin	NRP + Polyketide:Iterative type I polyketide + Polyketide:Enediyne type I polyketide	13%/6%	Both
6.1	NRPS-like,T1PKS	1	37,77	quinolidomicin A	Polyketide	28,%/67%	Both
2.1	RiPP-like	136,664	147,482	lymphostin/neolymphostinol B/lymphostinol/neolymphostin b	Polyketide + NRP	15%	MinION
2.5	NI-siderophore	4,182,055	4,194,725	peucechelin	NRP	10%	MinION
3.2	NRPS-like	407,718	448,468	sarpeptin A/sarpeptin B	NRP	25%	MinION
3.4	oligosaccharide, terpene	530,94	565,187	brasilicardin A	Terpene + Saccharide	38%	MinION
3.5	T2PKS,NRPS-like	732,357	804,659	pradimicin-A	Polyketide	17%	MinION
3.7	NRPS	1,520,332	1,559,968	bleomycin A2/bleomycin B2	NRP + Polyketide + Saccharide	14%	MinION
3.9	T1PKS	1,799,831	1,841,948	rakicidin A/rakicidin B	NRP:Cyclic depsipeptide + Polyketide:Modular type I polyketide	40%	MinION
3.10	NI-siderophore	1,859,147	1,870,220	putrebactin/avaroferrin	Other	50%	MinION

^a^ Similarity data from Illumina and MinION, respectively.

The type-III polyketide Loseolamycin was identified from *Micromonospora* endolithica and inhibited the growth of the Gram-positive *Bacillus subtilis* and also showed herbicidal activity against the weed Agrostis stolonifera (Lasch et al., 2020). Cinerubins are anthracycline antimicrobials produced by actinomycetota which also present antitumor activity (Paderog et al., 2020; Silva et al., 2020). BRA006 possesses highly similar clusters to cinerubin B (74% by MinION and 80% by Illumina) and loseolamycin (88% by MinION and 92% by Illumina) A1/A2. BRA006 also has less similar BGCs for the biosynthesis of other natural compounds with antitumoral activity such as bleomycins and kedarcidin, isolated from *Streptomyces* verticillus and an unclassified Actinomycetales strain (ATCC 53650) Actinomycetes, respectively (Hecht, 2000; Hofstead et al., 1992).

AntiSMASH results from Illumina assembly yielded 18 BGCs (Table 2) in total, including clusters for the production of quinolidomicyn, cinerubin B, and loseolamycin A1/A2 found in both Illumina and MinION data. However, only by sequencing with Illumina, it was possible to find a BGC 100% similar to the production of sungeidines (Low et al., 2020), a group of metabolites produced by pathways with close evolutionary relations with the antitumor dynemicins (Unno et al., 1997).

### 3.3 Evolutionary relationships

**FIGURE 2:**
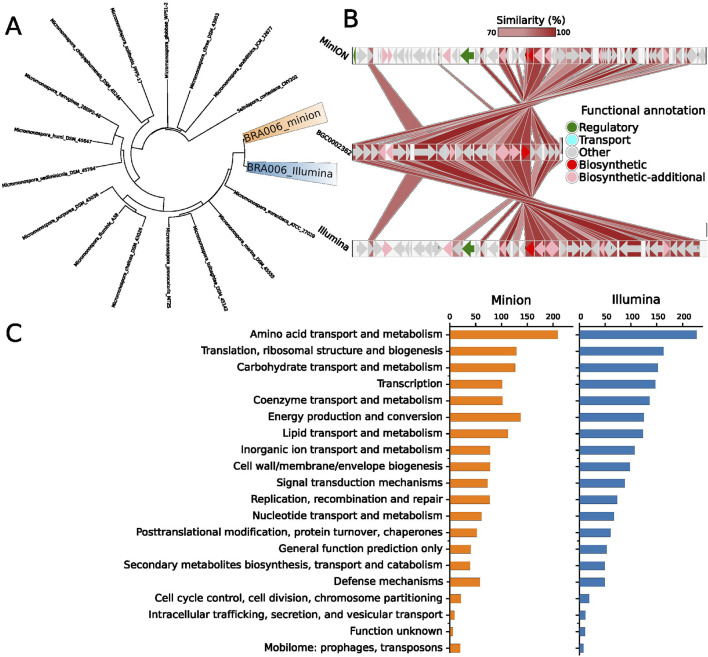
**(A)** Digital DNA:DNA hybridization phylogenetic tree of the Illumina and MinION data from the isolate BRA006. The Type Genome Server displays the 16 closest related genomes present in its database based on the genomic distance of the whole genome sequencing data. **(B)** Protein BLASTp similarity between BRA006 Loseolamycin BGC and reference BGC0002362 pointed by antiSMASH. All possible matches for every BRA006 match were filtered by the smallest e-value (See complete BLAST data in Supplementary Tables 1A, B). **(C)** The distribution of CDS annotated by Prokka according to Cluster of Orthologous Groups.

Since both genome sequencing methods yielded identical clusters with enzymes from pathways for synthesizing compounds with medical applications, we decided to explore the evolutionary relationship. According to digital DNA:DNA hybridization used by Type Genome Server (TYGS) (Meier-Kolthoff and Göker, 2019) for phylogenetic inferencing, these two assemblies were classified as *Micromonospora sp* and pointed out as possible novel species due to their relatively high genomic distance to its closest related group: *Micromonospora aurantiaca* ATCC 27029 (Figure 2). However, the BGC with higher similarity to the antiSMASH database is the one for the production of loseolamycin A1/A2 in both MinION and Illumina assemblies. Therefore we used BLASTp to compare the proteins within those BGCs to the reference: BGC0002362 from *Micromonospora endolithica.*

Figure 2B shows the protein similarity between loseolamycin-producing BGC from BRA006 and M. endolithica. Both assembly methods detected the complete inversion of this M. endolithica BGC followed by a series of indels in the upstream region majorly composed of CDSs that encode proteins with no functional annotation. Other BGCs with less similarity to the antiSMASH database were also compared with the reference sequences. For the cinebubin B and quinalidomicin A BGCs there were differences between antiSMASH results regarding the sequencing method. In the case of cinerubin B BGC, sequencing by Illumina resulted in a BGC with 172 Mb length while MinION’s with 71 Mb. Both with a few highly similar proteins to the reference BGS (BGC0000212) (Supplementary Figure 2) original from *Streptomyces sp*. SPBO074. On the other hand, quinolidomicin A BGCs showed 219 Mb in MinION and 70 Mb in Illumina assembly. Quinolidomicin A BGC from MinION data presented more similarity (67% against 45% from the counterpart) to reference BGC0002520 original from *Micromonospora sp* (Supplementary Figure 3) although with shorter CDSs.

### 3.4 Metabolomic analysis

**FIGURE 3:**
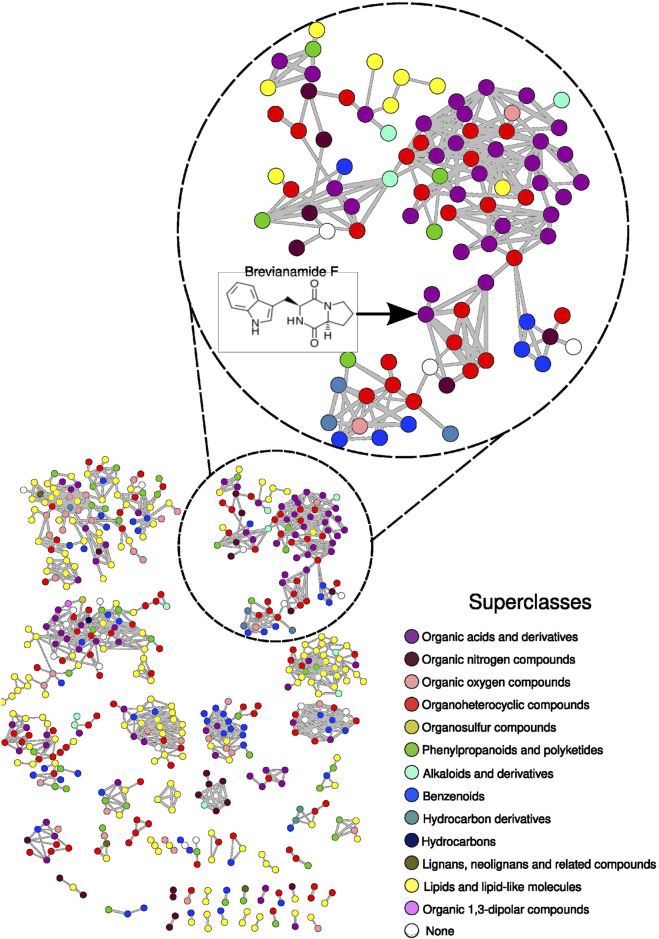
Molecular network constructed from BRA006 metabolomic data. Nodes were colored based on the superclass classification performed on the molecules annotated by ClassyFire. The full description of the annotation set is presented in Supplementary Table 4. The cluster showing the node with the annotation for brevianamide F is highlighted.

From the analysis of potential BGCs found in BRA006, where their potential to biosynthesize compounds with antibacterial (loseolamycins A, quinolidomicin A, cinerubin B, and brasilicardin A), antifungal (pradimicin A) and anticancer (bleomycin A2 and kedarcidin) activity was identified, we investigated whether these compounds were present in the metabolome of this Actinomycetota. To do so, and to extend the analysis to other compounds that BRA006 might be able to produce, we performed a metabolomics analysis in which the BRA006 extract was analyzed by LC-ESI(+)-HRMS/MS. After the LC-MS/MS analysis, the obtained raw data were converted into. mzXML format using Proteowizard (Chambers et al., 2012), and feature finding was performed using MZmine (Schmid et al., 2023). We then performed the annotations sequentially, in three steps. In the first, the annotations were made by spectral pairing and molecular network construction through GNPS2 (https://gnps2.org/). As a result, we obtained 527 nodes with at least one connection, of which 83 were annotated by GNPS2. Based on this result, we propagated the annotations to the nodes that did not present an annotation by spectral pairing using ChemWalker (Borelli et al., 2023), increasing the annotations to 373 nodes. Finally, for the nodes that could not be annotated by this tool, we used a third *in silico* spectral annotation tool, SIRIUS (Dührkop et al., 2019), increasing the annotations for an additional 67 nodes. Thus, a total of 527 nodes were represented by the molecular network, of which 523 were annotated. With these results in hand, we performed an automated chemical classification of each annotated compound using ClassyFire (Djoumbou Feunang et al., 2016). The molecular network was colored based on the superclasses to which each annotated compound belonged (Figure 3).

The compounds could be grouped into 13 different chemical superclasses, where 30.2% of the annotated compounds belong to the superclass of lipids and lipid-like molecules, 19.5% to organoheterocyclic compounds, 15.1% to organic acids and derivatives, 9.9% to benzenoids, 7.6% to phenylpropanoids and polyketides, 6.5% to organic oxygen compounds, 4.0% to organic nitrogen compounds, 1.7% to alkaloids and derivatives, 0.6% to lignans, neolignans, and related compounds, 0.4% to hydrocarbon derivatives, 0.2% to organic 1, 3-dipolar compounds, 0.2% to hydrocarbons, 0.2% to organosulfur compounds, and 3.8% could not be classified (None). The complete relationship between all annotated compounds and their chemical hierarchical classification into kingdom, superclass, and class is shown in Supplementary Table 4. From the entire set of annotations and classifications, we searched for the seven compounds predicted by antiSMASH in the BRA006 metabolome. None of the seven compounds were found in the annotation pool. Therefore, we took the molecular structure of these seven compounds and performed their chemical classification using ClassyFire; once we had their chemical class, we would search for compounds annotated in the BRA006 metabolome that had the same chemical class. From a pharmacological point of view, compounds belonging to the same chemical class might belong to the same biosynthetic pathway and or have similar effects, as is the case, for example, with the class of peptidomimetics in the treatment of cancer (de Valk et al., 2020) and steroids in the treatment of pain (Paulsen et al., 2013). The seven compounds were organized into six different classes of molecules, where loseolamycin A1 belongs to the phenol class, cinerubin B belongs to the anthracycline class, brasilicardin A belongs to the steroid and steroid derivative class, pradimicin A belongs to the naphthacene class, bleomycin A2 belongs to the peptidomimetic class, and quinolidomycin A and kedarcidin are organooxygen compounds.

Of these six chemical classes to which the compounds of interest belong, the Phenols class presented 10 annotated molecules, while the Organooxygen Compounds and Steroids and Steroid Derivatives classes presented 34 compounds each and Peptidomimetics presented two compounds in our analysis. Among the 10 compounds belonging to the Phenol class, three of them had bioactivity previously reported, but with a different action from the antibiotic loseolamycins A1. The targeting offered by antiSMASH, by searching for molecules belonging to the same class as those with bioactive activity predicted by the tool, allowed us to find a larger and more diverse range of compounds.

**FIGURE 4:**
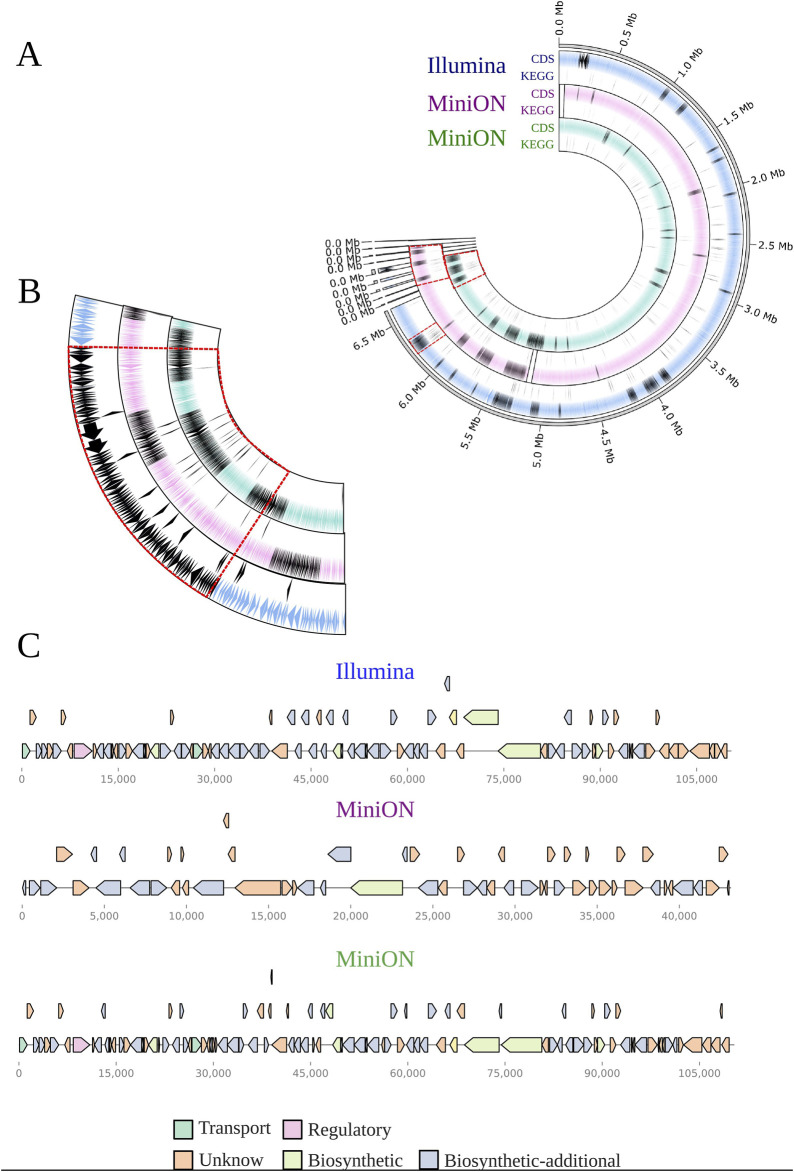
Circular representation of BRA006 genome according to the sequencing technique. **(A)** Each circle contains a track with the CDS predicted by prokka with BGCs annotated by AntiSMASH and a underneath track highlighting the proteins present in the KO00404 map from KEGG (https://www.genome.jp/pathway/ko00404). The pink-colored track contains results from MinION assembly pipeline with Guppy and Fly, whilst the green tracks refers to pipeline with Dorado and Unicyler. **(B)** Comparison among BGC. We set BGC number 20 from Illumina data as que reference to show the fragmented BGCs from MinION data. **(C)** Detailed visualization of Illumina BGC 20 and their respective corresponding from MinION.

Using a reverse flow of integrative analysis, where we start from what was annotated in the metabolome, we set out to evaluate whether it would be possible to identify, from a given metabolite, the enzymes that lead to its production in BRA006. To this end, we first crossed the annotated metabolome with the KEGG database (Kanehisa and Goto, 2000) to search within the metabolome of this Actinomycetota for molecules with known biosynthetic pathways. As a result, we found a molecule belonging to the staurosporin biosynthetic pathway, brevianamide F. It should be noted that of the three methods used in the annotation process, brevianamide F was annotated by spectral pairing with the GNPS spectral library (Wang et al., 2016), showing a MQScore of 0.97 and a m/z error of 2.47ppm. Since the topology of the molecular network is given by the similarity between nodes, the node with an annotation for brevianamide F is connected to seven other nodes (Figure 3). All seven nodes have been annotated by ChemWalker. Looking at the predicted structures, six of the seven share the same indole-like nucleus as brevianamide F, suggesting that other molecules may ultimately be produced, either by brevianamide F BGC or by other intermediates belonging to the same pathway as brevianamide F. Once identified, the enzymes that make up the staurosporin biosynthetic pathway, we searched the BRA006 genome for which of these enzymes would be encoded. From this search, 16 staurosporin pathway enzymes were identified in the genome of this Actinomycetota (Supplementary Figure 4), and 11 overlap the region of the BGC 20 from Illumina (Figure 4). Among them, we found an NRPK 2,3-dihydroxybenzoate-AMP ligase functionally classified as a biosynthetic-additional enzyme by antiSMASH. These results show that a dynamic integrative approach, i.e., first combining spectral and *in silico* annotation, assigning chemical classes, and then searching for metabolites from the genome to match encoded proteins in the genome to the pathway producing the putative metabolite annotated, is an efficient approach to characterize new species with the potential to produce bioactive compounds.

## 4 Discussion

The multiomics characterization of new *Micromonospora* strains, especially those found in the marine environment, is a highly relevant task given the potential for the discovery of new bioactive compounds. Multiomics approaches allow combining information from different sources and amplify the scope of features that can be learnt (Kwoji et al., 2023). For instance, genomic annotation are hypothesis on the proteins that a living organism can produce, although there is no information about the levels nor the conditions in which these proteins are expressed. On the other hand, metabolomics inform the set of compounds conditionally produced but without information on the enzymes or metabolic pathways (O’Donell et al., 2020). Metabologenomics, integrates genomic and metabolomics and relate the compounds found to the enzymes encoded in the genome (Go et al., 2024).

In this sense, the use of dynamic integrative analytical approaches seems to be a promising resource, both in the process of characterizing new species and in the evaluation of the potential for the production of natural products from a new microorganism.

In the present work, we characterized BRA006, a potential new species of the genus *Micromonospora* in terms of secondary metabolites production. We used both genomic and metabolomic points of view, comparing two whole genome sequencing approaches (Figure 2A) and a multiple-step metabolite annotation workflow. This strategy introduces an innovative dynamic approach to multiomics analysis in which the annotated BGCs types predicted by antiSMASH guided the search for bioactive compounds in the metabolome content, as well as the compounds identified in the metabolome (which match specific pathways) led to the search for CDSs that encode enzymes from these pathways. Among the various microbial genera described to date that stand out for their ability to produce bioactive compounds, the genus *Micromonospora* is an important model in natural products research and a milestone in the discovery of new biocompounds (Hifnawy et al., 2020). The potential to produce bioactive natural products from bacterial isolates from the Brazilian coast is already known, especially those with antitumor activity (Sousa et al., 2012; Silva et al., 2017). Among these, the activity observed in crude extracts of the genus *Micromonospora* was attributed to a group of anthracyclinones (Sousa et al., 2012).

According to Yan et al. (2022), species from the *Micromonospora* genus encode from 4,200 to 8,017 proteins and have genome sizes from 5.07 to 9.24 Mb. BRA006 possesses a 6.7 Mb genome, confirmed by two independent sequencing methods, although MinION assembly annotated by Prokka showed up to 11.000 CDS against 6,080 from Illumina. This gap between them is mostly due to higher error rates from MinION assembly, which probably causes artificial stop codons that could explain the higher number of CDSs. We decreased this gap changing our basecaller and genome assembler tools from Gumpy to Dorado and from Flyer to Unicycler, respectively. However, this still resulted in a fragmented kedarcidin-producing BGC which overlaps with the genes that encode enzymes that are also part of the staurosporin pathway. An alternative to solve this issue would be to perform a hybrid assembly (Wick et al., 2017b; Laver et al., 2015). Besides the difference in CDS number, antiSMASH found the same BGCs in both assemblies, such as Cinerubin B and Loseolamycins, with high similarity to antiSMASH database. Also, the whole genome sequence phylogeny placed both assemblies as a monophyletic group dissimilar enough from *Micromonispora* aurantiaca ATCC27029 to TYGS to point BRA006 as a possible novel *Micromonospora* species. As an example of genetic divergence of BRA006 from other *Micromonospora*, we can cite the loseolamycin A1 BGC, where it is possible to observe a complete inversion of the cluster and several indels. According to Medema et al. (2014) NRPK clusters evolved from gene duplication followed by differentiation, which could explain the difference between BRA006 and M. endolithica. Unfortunately, even with Prokka, annotating most of the proteins in that BGC was not yet possible.

The approach “from genomics to metabolomics” yielded a BGC that encodes pathways to compounds with pharmaceutical applications, which confirms the importance of the *Micromonospora* genus, although the compounds produced by these BGCs were not found in metabolomic data. Their absence can be explained by differences between laboratory culture media and the original ecological niche, as well as the need for improvements in the acquisition parameters. Genomic-guided works often require heterologous expression of parts or the entire BGC to obtain the active compound in the laboratory (Xu and Wright, 2019). For instance, Lasch et al. (2020) obtained loseolamycins from M. endolithica by heterologously expressing type III polyketide synthase, Domingues Vieira et al. increased the production of eponemycin and related epoxyketone peptides by cloning the whole epn-tmc BGC from *Streptomyces* sp. BRA346 (Domingues Vieira et al., 2022), and Yamanaka produced Taraomycin A by editing regulators of this BGC from *Saccharomonospora* sp. CNQ490 (Yamanaka et al., 2014).

Starting from metabolomics, the analysis of the BRA006 metabolome allowed us to identify annotated compounds belonging to already well-established chemical classes whose biosynthesis is reported in the literature for this genus, as in the case of macrolides (Hifnawy et al., 2020). Of these, we were able to annotate nine compounds belonging to this class (Supplementary Table 4), two of which previously reported bioactivity: tricholide A, with antibacterial activity (Bertin et al., 2017) and 11,12-dihydroxy-6,14-dimethyl-1,7-dioxacyclotetradeca-3,9-diene-2,8-dione, with immunosuppressive activity (Fujimoto et al., 1998). It should be noted that two compounds were recorded as tricholide A. Both presented the same m/z value but with very different retention times, indicating the presence of isomers of this compound, as both appear as neighboring nodes in the molecular network, and also which can be seen by the extratec ion cromatogram (XIC) (Supplementary Figure 5B). The annotation procedures and KEGG pathway search identified Brevianamide F, a compound with activity against *Staphylococcus aureus* (Ben Ameur Mehdi et al., 2009) and an intermediate of well-established inducer of apoptosis, Staurosporin (Belmokhtar et al., 2001). Among the three annotation methods for a given molecule we used, spectral matching against a reference library is the best available resource (Ausloos et al., 1999). In addition, we used two other *in silico* annotation resources: ChemWalker and SIRIUS. Of these two tools, SIRIUS has the best accuracy, but it is very difficult to use when dealing with large sets of spectra. This limitation is overcome by ChemWalker, which allows greater annotation coverage, taking into account the topology of the molecular network. We reached brevianamide F annotation through two different annotation routes, which brings robustness to the interpretation of the result obtained and highlights the potential for a reverse flow in elucidating the biosynthetic potential of a new non-model organism. The spectral library match was reached through pairing spectra on GNPS2, where the results on XIC as well as the mirrorplot for this annotated compound is presented in Supplementary Figure 5. It is worth highlighting that we also carried out an *in silico* prediction analysis using SIRIUS for the seven nodes related to that of Brevianimide F. However, the best candidates predicted by this tool had little structural similarity with the compound itself, unlike the candidates provided by ChemWalker. The advantage of ChemWalker is that it uses the sample context given by the molecular network topology on compound re-ranking. Eventually, this new proposed BGC could synthesize the other molecules annotated and linked to the brevianamide F’s node or, maybe, they are intermediates from different pathways that had not been described yet. In both cases, the knowledge of these analogues opens the possibilities to improve the known bioactivity of Brevianamide F, which could be tested by isolation or even by (bio)synthesizing the compounds.

Inspecting KEGG’s metabolic pathways, we found that brevianamide F, a product of fungi metabolism (Mehetre et al., 2019), is part of staurosporin biosynthesis (KO00404). Therefore, we retrieved all EC numbers from the KO00404 pathway, connected them to our Prokka data, and found three enzymes that can catalyze the Brevianamide F synthesis reaction. In the KO00404 pathway, brevianamide F biosynthesis requires tryptophan and proline as substrates, being its core assembled by a non-ribosomal peptide synthetase (COG1020), named brevianamide F synthase (EC: 6.3.2.-), which is encoded by the gene FtmA (NCBI ID Aspergillus fumigatus (AFUA_8G00170) (Wang et al., 2023), and is also reported in *Streptomyces* sp (Maiya et al., 2006). The isolate BRA006 has an NRPK mbtB_1 (MinION data) that matches with COG and EC number of FtmA, but is not a component of any BGC found by antiSMASH. However, examining the downstream and upstream regions of 2,3-dihydroxybenzoate-AMP ligase CDS (Illumina data) we found a genomic region that has the potential to be part of the brevianemide F synthesis pathway BGC.

AntiSMASH identifies BGCs based on profiles of Hidden Markov Models (pHMM) from PFAM (Mistry et al., 2021), TIGRFAMs (Haft et al., 2013), SMART (Letunic et al., 2021), BAGEL (van Heel et al., 2018; Yadav et al., 2009) and custom models that recognize signature sequences of such conserved domains in genomic query sequences (Biermann et al., 2022). However, there are BGCs that lack universal class-specific signature sequences and therefore are partially identified. To overcome this limitation, deep-learning-based tools such as DeepBGC (Hannigan et al., 2019) have been applied in genomic mining research to uncover new BGCs.

It is interesting to emphasize the innovative approach used in the present work with a two-way analysis of the genome and metabolome. We started with the metabolome to see if the biosynthetic gene clusters involved in the production of a given metabolite could be identified in the genome. We then analyzed the genome sequencing data, and from there, by searching specific databases such as antiSMASH, we went to the metabolome to check whether the compounds predicted by antiSMASH were being produced (Domingues Vieira et al., 2022). Traditionally, the search for potential new compounds with bioactivity follows the latter linear flow of analysis, which in our case did not result in the identification of 7 of the metabolites predicted by genome mining. However, by using the chemical classes of these compounds, we could find analogues in our metabolomic data.

In addition, we present a new approach that integrates the classical approach with a reverse analysis, starting from the metabolome to the genome. For example, in neither short-read (Illumina) nor long-read (minION) sequencing data, it was possible to automatically detect the biosynthetic gene cluster for brevianamide F or staurosporin production. By using the two-way approach we could identify brevianamide F (reported as an intermediate in the Staurosporin biosynthesis) in BRA006 metabolome and, from there, we could identify some of brevianamide F′ putative analogs and a BGC in the BRA006 genome, initially annotated with other function, that could represent Brevianamide F biosynthetic pathway in BRA006. Therefore, the approach presented in the present work allowed us to extend the characterization of the potential of bioactive natural products produced by BRA006.

## Data Availability

All mass spectrometry metabolomics data are available at MassIVE through the identifier: MSV000095044. The parameters used for preprocessing in MZmine3 are available at Zenodo https://doi.org/10.5281/zenodo.10366840. All data, Python scripts and Jupyter notebooks used during multiomics data analysis are available on this project GitHub page: https://github.com/computational-chemical-biology/metabologenomics. The FBMN results can be found here https://gnps2.org/status?task=7b134da60f0f4a80aec790d2a294aedd and ChemWalker results here https://gnps2.org/status?task=9141a5cdabf842d39387e514e5305398. The assemblies are available on NBCI database. MiION: https://www.ncbi.nlm.nih.gov/biosample/SAMN39609461/ and Illumina: https://www.ncbi.nlm.nih.gov/biosample/?term=SAMN29586427.
