# Supplementary material for: A multi-omics reciprocal analysis for characterization of bacterial metabolism
Source: Front Mol Biosci. 2025 Mar 20;12:1515276. doi: 10.3389/fmolb.2025.1515276 (PMC11965639; doi:10.3389/fmolb.2025.1515276)
Supplement: Supplementary file 1 [file Table1.docx]

Supplementary Table 1. Results from BLAST between BRA006 Loseolamycin BGC and MiBiG reference BGC0002362 for both sequencing approaches.

**A: MiniON**

| Query ID | Subject ID | Protein Identity (%) | Alignment Length | Mismatch | Gaps | Query Start | Query End | Subject Start | Subject End | E-value | Bitscore |
| --- | --- | --- | --- | --- | --- | --- | --- | --- | --- | --- | --- |
| ONHDLDEL_03010 | LU17765_001620 | 83.333 | 6 | 1 | 0 | 7 | 12 | 595 | 600 | 7.9 | 18.1 |
| ONHDLDEL_03023 | LU17765_001740 | 85.714 | 7 | 1 | 0 | 9 | 15 | 124 | 130 | 2.1 | 16.9 |
| ONHDLDEL_03030 | LU17765_001780 | 95.27 | 148 | 7 | 0 | 1 | 148 | 1 | 148 | 1.39E-101 | 280 |
| ONHDLDEL_03033 | LU17765_001770 | 90.411 | 219 | 21 | 0 | 1 | 219 | 1 | 219 | 4.42E-123 | 341 |
| ONHDLDEL_03034 | LU17765_001760 | 100 | 106 | 0 | 0 | 1 | 106 | 56 | 161 | 1.01E-73 | 207 |
| ONHDLDEL_03036 | LU17765_001750 | 85 | 100 | 15 | 0 | 1 | 100 | 132 | 231 | 1.27E-39 | 123 |
| ONHDLDEL_03038 | LU17765_001730 | 90 | 20 | 2 | 0 | 11 | 30 | 329 | 348 | 1.48E-08 | 38.5 |
| ONHDLDEL_03039 | LU17765_001730 | 81.176 | 85 | 16 | 0 | 1 | 85 | 244 | 328 | 3.79E-36 | 116 |
| ONHDLDEL_03040 | LU17765_001730 | 92.857 | 182 | 13 | 0 | 3 | 184 | 25 | 206 | 7.49E-117 | 328 |
| ONHDLDEL_03041 | LU17765_001720 | 94.408 | 304 | 17 | 0 | 1 | 304 | 81 | 384 | 0 | 536 |
| ONHDLDEL_03042 | LU17765_001710 | 90.873 | 252 | 22 | 1 | 1 | 252 | 1 | 251 | 2.04E-159 | 434 |
| ONHDLDEL_03045 | LU17765_001700 | 77.419 | 93 | 18 | 1 | 1 | 90 | 185 | 277 | 3.52E-22 | 79 |
| ONHDLDEL_03046 | LU17765_001690 | 71.084 | 249 | 70 | 2 | 20 | 267 | 1 | 248 | 2.8E-103 | 293 |
| ONHDLDEL_03047 | LU17765_001680 | 77.433 | 483 | 88 | 4 | 34 | 505 | 10 | 482 | 0 | 662 |
| ONHDLDEL_03048 | LU17765_001670 | 91.729 | 399 | 30 | 3 | 1 | 399 | 1 | 396 | 0 | 696 |
| ONHDLDEL_03049 | LU17765_001660 | 84.746 | 59 | 9 | 0 | 1 | 59 | 195 | 253 | 3.79E-36 | 113 |
| ONHDLDEL_03050 | LU17765_001650 | 92.92 | 113 | 8 | 0 | 1 | 113 | 75 | 187 | 1.25E-75 | 214 |
| ONHDLDEL_03051 | LU17765_001650 | 83.333 | 66 | 11 | 0 | 1 | 66 | 1 | 66 | 2.75E-35 | 110 |
| ONHDLDEL_03052 | LU17765_001640 | 93.75 | 80 | 5 | 0 | 1 | 80 | 91 | 170 | 3.46E-48 | 142 |
| ONHDLDEL_03053 | LU17765_001640 | 90.909 | 88 | 8 | 0 | 1 | 88 | 1 | 88 | 7.91E-42 | 127 |
| ONHDLDEL_03054 | LU17765_001630 | 89.831 | 59 | 6 | 0 | 13 | 71 | 25 | 83 | 1.28E-32 | 108 |
| ONHDLDEL_03055 | LU17765_001630 | 93.293 | 164 | 11 | 0 | 1 | 164 | 136 | 299 | 1.76E-100 | 284 |
| ONHDLDEL_03057 | LU17765_001620 | 99.115 | 113 | 1 | 0 | 1 | 113 | 484 | 596 | 2.88E-75 | 230 |
| ONHDLDEL_03059 | LU17765_001620 | 100 | 28 | 0 | 0 | 1 | 28 | 344 | 371 | 4.75E-16 | 61.2 |
| ONHDLDEL_03060 | LU17765_001620 | 100 | 224 | 0 | 0 | 1 | 224 | 87 | 310 | 1.94E-153 | 440 |
| ONHDLDEL_03061 | LU17765_001610 | 96.364 | 55 | 2 | 0 | 1 | 55 | 62 | 116 | 3.75E-35 | 106 |
| ONHDLDEL_03062 | LU17765_001600 | 97.561 | 123 | 3 | 0 | 1 | 123 | 380 | 502 | 2.59E-83 | 244 |
| ONHDLDEL_03063 | LU17765_001600 | 96.8 | 125 | 4 | 0 | 1 | 125 | 223 | 347 | 2.02E-84 | 249 |
| ONHDLDEL_03064 | LU17765_001600 | 85.333 | 225 | 21 | 3 | 1 | 217 | 1 | 221 | 3.73E-132 | 373 |
| ONHDLDEL_03065 | LU17765_001580 | 81.646 | 158 | 26 | 1 | 1 | 158 | 42 | 196 | 3.6E-88 | 252 |
| ONHDLDEL_03068 | LU17765_001560 | 79.913 | 229 | 41 | 2 | 1 | 226 | 71 | 297 | 9.83E-120 | 335 |
| ONHDLDEL_03069 | LU17765_001550 | 81.053 | 95 | 18 | 0 | 1 | 95 | 337 | 431 | 6.64E-50 | 155 |
| ONHDLDEL_03070 | LU17765_001550 | 93.671 | 158 | 10 | 0 | 1 | 158 | 173 | 330 | 4.72E-103 | 294 |

**B: Illumina**

| Query ID | Subject ID | Protein Identity (%) | Alignment Length | Mismatch | Gaps | Query Start | Query End | Subject Start | Subject End | E-value | Bitscore |
| --- | --- | --- | --- | --- | --- | --- | --- | --- | --- | --- | --- |
| OPPBIMDH_00886 | LU17765_001620 | 83.333 | 6 | 1 | 0 | 7 | 12 | 595 | 600 | 7.7 | 18.1 |
| OPPBIMDH_00896 | LU17765_001790 | 77.049 | 61 | 14 | 0 | 37 | 97 | 1 | 61 | 9.39E-21 | 77.8 |
| OPPBIMDH_00899 | LU17765_001780 | 93.75 | 160 | 10 | 0 | 1 | 160 | 1 | 160 | 1.86E-109 | 300 |
| OPPBIMDH_00902 | LU17765_001770 | 90.411 | 219 | 21 | 0 | 1 | 219 | 1 | 219 | 4.42E-123 | 341 |
| OPPBIMDH_00903 | LU17765_001760 | 100 | 161 | 0 | 0 | 1 | 161 | 1 | 161 | 2.41E-116 | 318 |
| OPPBIMDH_00904 | LU17765_001750 | 74.359 | 234 | 52 | 2 | 3 | 231 | 1 | 231 | 5.68E-75 | 219 |
| OPPBIMDH_00906 | LU17765_001730 | 91.092 | 348 | 30 | 1 | 1 | 348 | 1 | 347 | 0 | 601 |
| OPPBIMDH_00907 | LU17765_001720 | 94.072 | 388 | 23 | 0 | 1 | 388 | 1 | 388 | 0 | 662 |
| OPPBIMDH_00908 | LU17765_001710 | 90.873 | 252 | 22 | 1 | 1 | 252 | 1 | 251 | 2.04E-159 | 434 |
| OPPBIMDH_00909 | LU17765_001700 | 84.364 | 275 | 40 | 1 | 5 | 276 | 3 | 277 | 1.72E-114 | 323 |
| OPPBIMDH_00910 | LU17765_001690 | 72.177 | 248 | 64 | 3 | 1 | 247 | 1 | 244 | 6.85E-106 | 299 |
| OPPBIMDH_00911 | LU17765_001680 | 96.058 | 482 | 19 | 0 | 2 | 483 | 1 | 482 | 0 | 899 |
| OPPBIMDH_00912 | LU17765_001670 | 95.729 | 398 | 15 | 2 | 1 | 398 | 1 | 396 | 0 | 735 |
| OPPBIMDH_00913 | LU17765_001660 | 71.595 | 257 | 69 | 2 | 1 | 257 | 1 | 253 | 4.56E-107 | 302 |
| OPPBIMDH_00914 | LU17765_001650 | 89.84 | 187 | 19 | 0 | 1 | 187 | 1 | 187 | 2.17E-122 | 335 |
| OPPBIMDH_00915 | LU17765_001640 | 92.941 | 170 | 12 | 0 | 1 | 170 | 1 | 170 | 3.7E-98 | 273 |
| OPPBIMDH_00916 | LU17765_001630 | 91.973 | 299 | 24 | 0 | 6 | 304 | 1 | 299 | 0 | 529 |
| OPPBIMDH_00917 | LU17765_001620 | 99.408 | 845 | 5 | 0 | 1 | 845 | 1 | 845 | 0 | 1681 |
| OPPBIMDH_00918 | LU17765_001610 | 94.828 | 116 | 6 | 0 | 1 | 116 | 1 | 116 | 7.06E-62 | 177 |
| OPPBIMDH_00919 | LU17765_001600 | 95.618 | 502 | 22 | 0 | 1 | 502 | 1 | 502 | 0 | 974 |
| OPPBIMDH_00920 | LU17765_001580 | 80.321 | 249 | 45 | 2 | 1 | 248 | 1 | 246 | 1.62E-140 | 386 |
| OPPBIMDH_00921 | LU17765_001570 | 95.238 | 252 | 12 | 0 | 1 | 252 | 1 | 252 | 2.68E-172 | 467 |
| OPPBIMDH_00922 | LU17765_001560 | 91.246 | 297 | 26 | 0 | 1 | 297 | 1 | 297 | 0 | 526 |
| OPPBIMDH_00923 | LU17765_001550 | 92.111 | 431 | 34 | 0 | 131 | 561 | 1 | 431 | 0 | 774 |
