# Supplementary material for: A multi-omics reciprocal analysis for characterization of bacterial metabolism
Source: Front Mol Biosci. 2025 Mar 20;12:1515276. doi: 10.3389/fmolb.2025.1515276 (PMC11965639; doi:10.3389/fmolb.2025.1515276)
Supplement: Supplementary file 2 [file Table2.docx]

Supplementary Table 2. Results from BLAST between BRA006 cinerubin-B BGC and MiBiG reference BGC0000212 for both sequencing approaches.

**A: MiniON**

| Query ID | Subject ID | Protein Identity (%) | Alignment Length | Mismatch | Gaps | Query Start | Query End | Subject Start | Subject End | E-value | Bitscore |
| --- | --- | --- | --- | --- | --- | --- | --- | --- | --- | --- | --- |
| ONHDLDEL_08762 | SSBG_00503 | 79.49 | 156 | 32 | 0 | 1 | 156 | 131 | 286 | 1.19E-90 | 258 |
| ONHDLDEL_08772 | SSBG_00505 | 76.79 | 56 | 13 | 0 | 1 | 56 | 34 | 89 | 4.27E-27 | 90.5 |
| ONHDLDEL_08773 | SSBG_00505 | 80 | 25 | 5 | 0 | 3 | 27 | 1 | 25 | 9.79E-11 | 44.7 |
| ONHDLDEL_08775 | SSBG_00490 | 83.33 | 6 | 1 | 0 | 23 | 28 | 308 | 313 | 2.7 | 15 |
| ONHDLDEL_08781 | SSBG_00501 | 71.93 | 57 | 16 | 0 | 4 | 60 | 51 | 107 | 7.06E-26 | 89 |
| ONHDLDEL_08784 | SSBG_00502 | 75.58 | 258 | 62 | 1 | 1 | 257 | 112 | 369 | 1.35E-135 | 378 |
| ONHDLDEL_08785 | SSBG_00502 | 81.31 | 107 | 20 | 0 | 1 | 107 | 1 | 107 | 1.97E-60 | 181 |
| ONHDLDEL_08789 | SSBG_00487 | 72.04 | 93 | 26 | 0 | 1 | 93 | 32 | 124 | 1.43E-45 | 139 |
| ONHDLDEL_08790 | SSBG_00487 | 76.86 | 121 | 27 | 1 | 1 | 120 | 134 | 254 | 1.93E-59 | 180 |
| ONHDLDEL_08803 | SSBG_00496 | 77.27 | 198 | 45 | 0 | 1 | 198 | 64 | 261 | 1.02E-114 | 319 |
| ONHDLDEL_08806 | SSBG_00494 | 80.77 | 26 | 5 | 0 | 1 | 26 | 391 | 416 | 3.58E-10 | 42.7 |
| ONHDLDEL_08807 | SSBG_00493 | 76.09 | 46 | 11 | 0 | 1 | 46 | 138 | 183 | 4.6E-24 | 82.8 |
| ONHDLDEL_08862 | SSBG_00493 | 87.5 | 8 | 1 | 0 | 65 | 72 | 261 | 268 | 2.8 | 18.5 |

**B: Illumina**

| Query ID | Subject ID | Protein Identity (%) | Alignment Length | Mismatch | Gaps | Query Start | Query  End | Subject Start | Subject End | E-value | Bitscore |
| --- | --- | --- | --- | --- | --- | --- | --- | --- | --- | --- | --- |
| OPPBIMDH_04960 | SSBG_00494 | 71.845 | 412 | 116 | 0 | 3 | 414 | 5 | 416 | 0 | 615 |
| OPPBIMDH_04962 | SSBG_00496 | 72.656 | 256 | 70 | 0 | 6 | 261 | 6 | 261 | 1.46E-140 | 387 |
| OPPBIMDH_04972 | SSBG_00487 | 75.785 | 223 | 54 | 0 | 1 | 223 | 32 | 254 | 3.55E-122 | 341 |
| OPPBIMDH_04976 | SSBG_00502 | 77.778 | 369 | 82 | 0 | 1 | 369 | 1 | 369 | 0 | 570 |
| OPPBIMDH_04981 | SSBG_00505 | 73.663 | 243 | 64 | 0 | 1 | 243 | 1 | 243 | 3.71E-131 | 363 |
| OPPBIMDH_04986 | SSBG_00503 | 77.273 | 286 | 65 | 0 | 1 | 286 | 1 | 286 | 1.77E-157 | 432 |
| OPPBIMDH_05015 | SSBG_00514 | 75 | 8 | 2 | 0 | 14 | 21 | 301 | 308 | 1.7 | 18.1 |
