# Supplementary material for: A multi-omics reciprocal analysis for characterization of bacterial metabolism
Source: Front Mol Biosci. 2025 Mar 20;12:1515276. doi: 10.3389/fmolb.2025.1515276 (PMC11965639; doi:10.3389/fmolb.2025.1515276)
Supplement: Supplementary file 3 [file DataSheet1.pdf]

Supplementary Material

Figures

A multi-omics reciprocal analysis for characterization of bacterial metabolism

Gabriel Santos Arini, Tiago Cabral Borelli, Elthon Góis Ferreira, Rafael de Felício, Paula Rezende-Teixeira, Matheus Pedrino, Franciene Rabiço, Guilherme Marcelino Viana de Siqueira, Luiz Gabriel Mencucin, Henrique Tsuji, Lucas Sousa Neves Andrade, Leandro Maza Garrido, Gabriel Padilla, Alberto Gil-de-la-Fuente, Mingxun Wang, Norberto Peporine Lopes, Daniela Barretto Barbosa Trivella, Letícia Veras Costa-Lotufo, María-Eugenia Guazzaroni, Ricardo Roberto da Silva

Supplementary Material

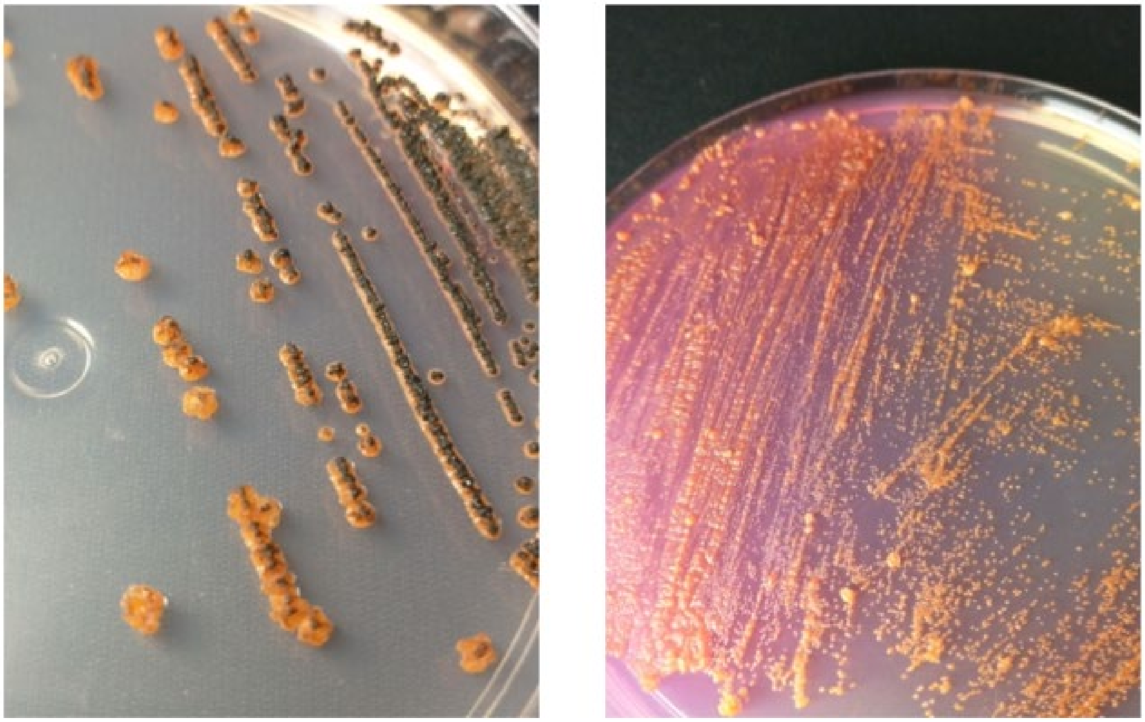

Supplementary Figure 1. Macroscopic aspects of BRA006 colonies. On the left, the colonies are highlighted in orange with their apical growth and their respective spores in black. On the right, the color of the water is highlighted by the growth of this bacterium.

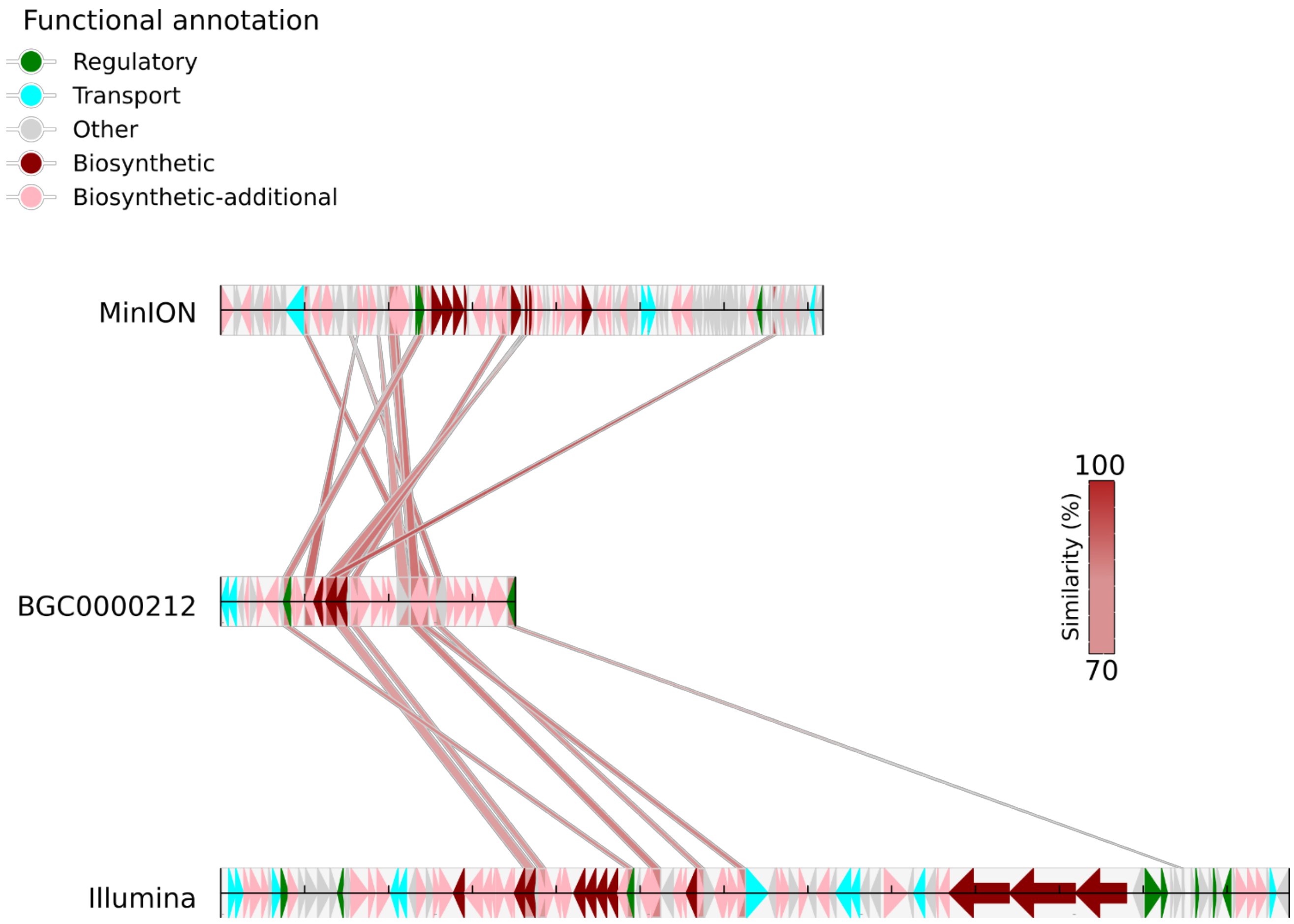

Supplementary Figure 2. Comparison between AntiSMASH results of cinerubin B-producing BGC from genomes sequenced by Illumina and MinION (with Guppy for base-calling and Flyer as assembler). The sequence similarity among BGC components is represented by strings attached to them. The redder the string, the more identical the two CDSs are. The complete results from BLAST between BRA006 annotated proteins and AntiSMASH BGCs reference are available in Supplementary Table 2.

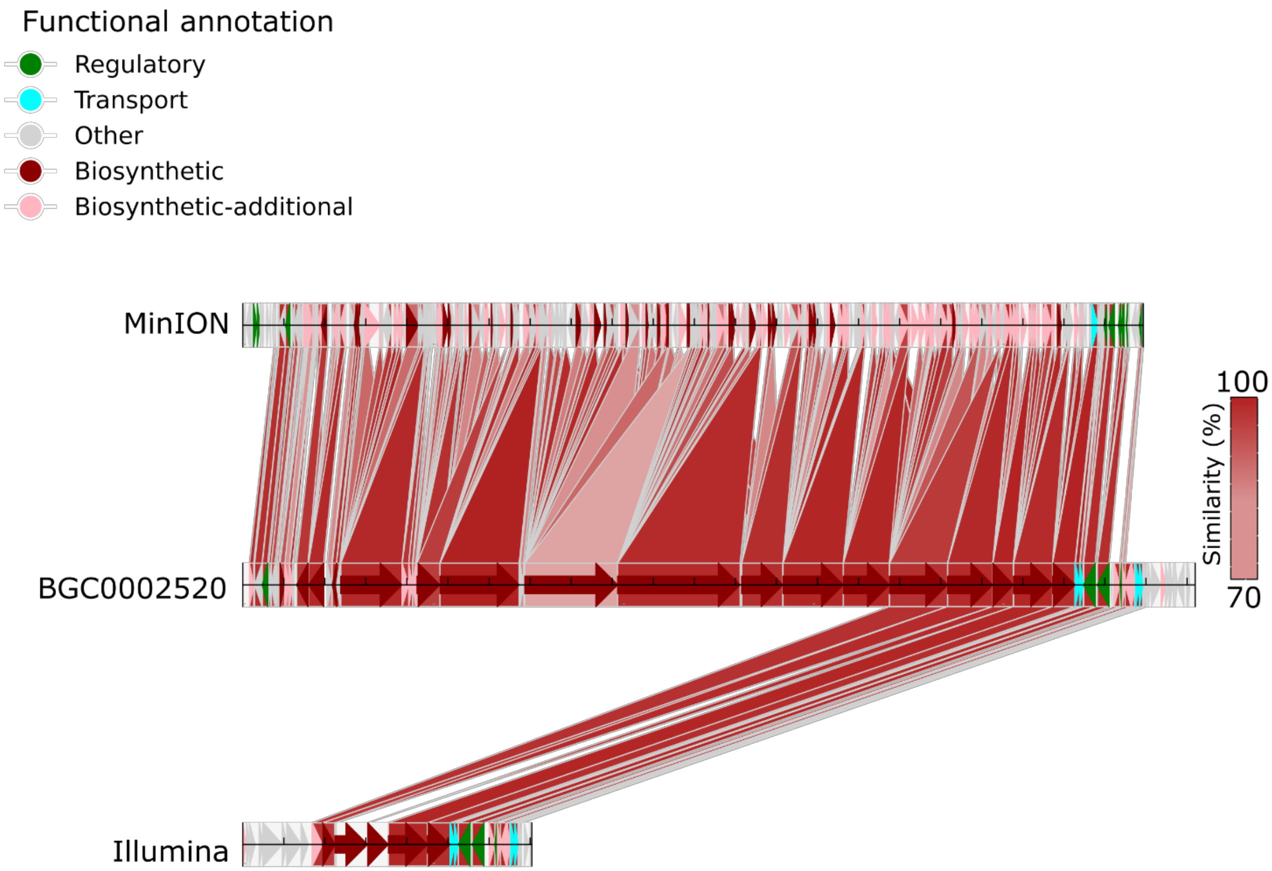

Supplementary Figure 3. Comparison between AntiSMASH results of Quinolidomicin A-producing BGC from genomes sequenced by Illumina and MinION (with Guppy for base-calling and Flyer as assembler). The sequence similarity among BGC components is represented by strings attached to them. The redder the string, the more identical the two CDSs are. The complete results from BLAST between BRA006 annotated proteins and AntiSMASH BGCs reference are available in Supplementary Table 3.

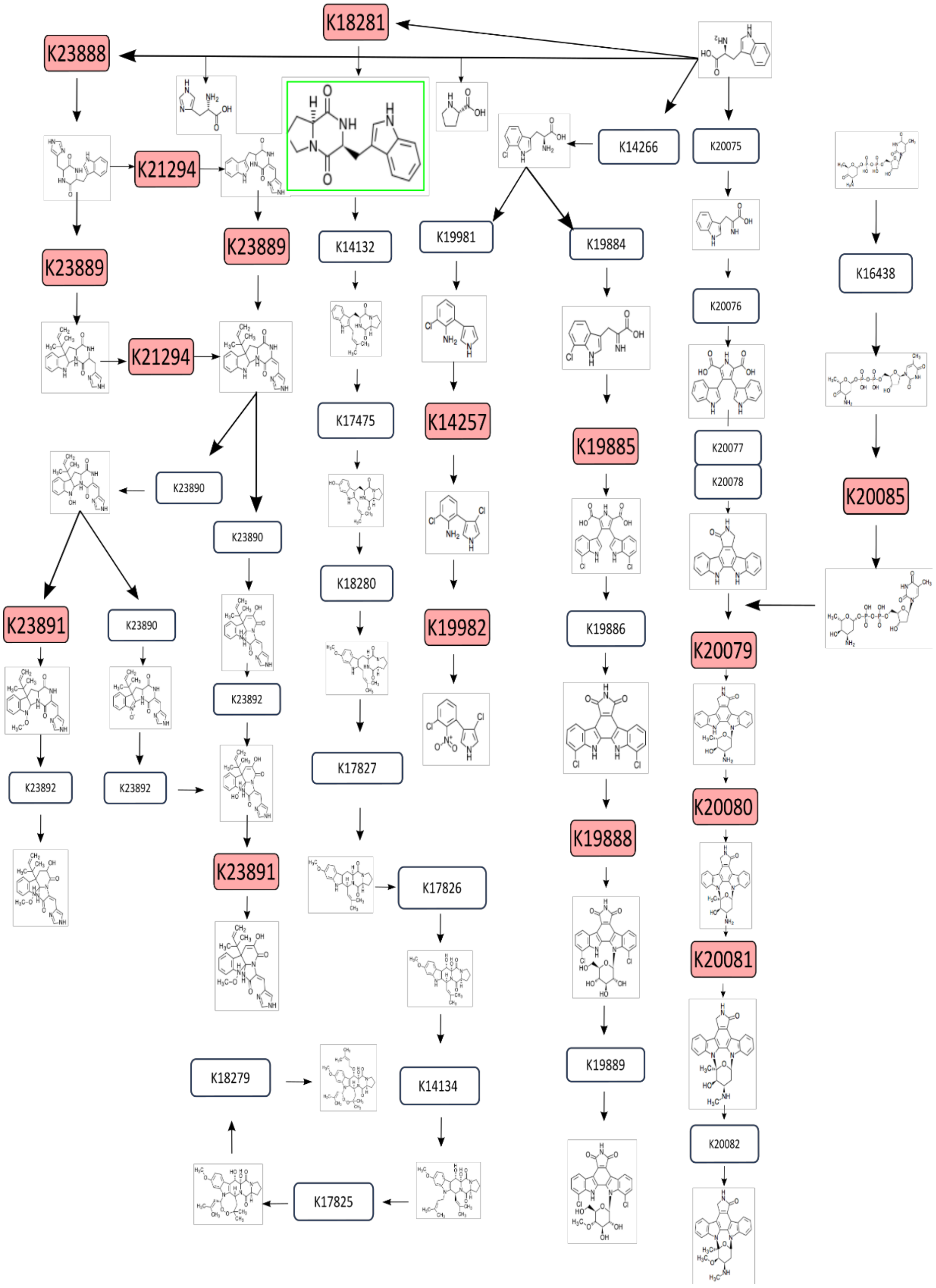

Supplementary Figure 4. Biosynthetic pathway of staurosporine. The highlighted boxes refer to the enzymes annotated in the BRA006 genome with the same EC number that staturosporin pathways enzymes.

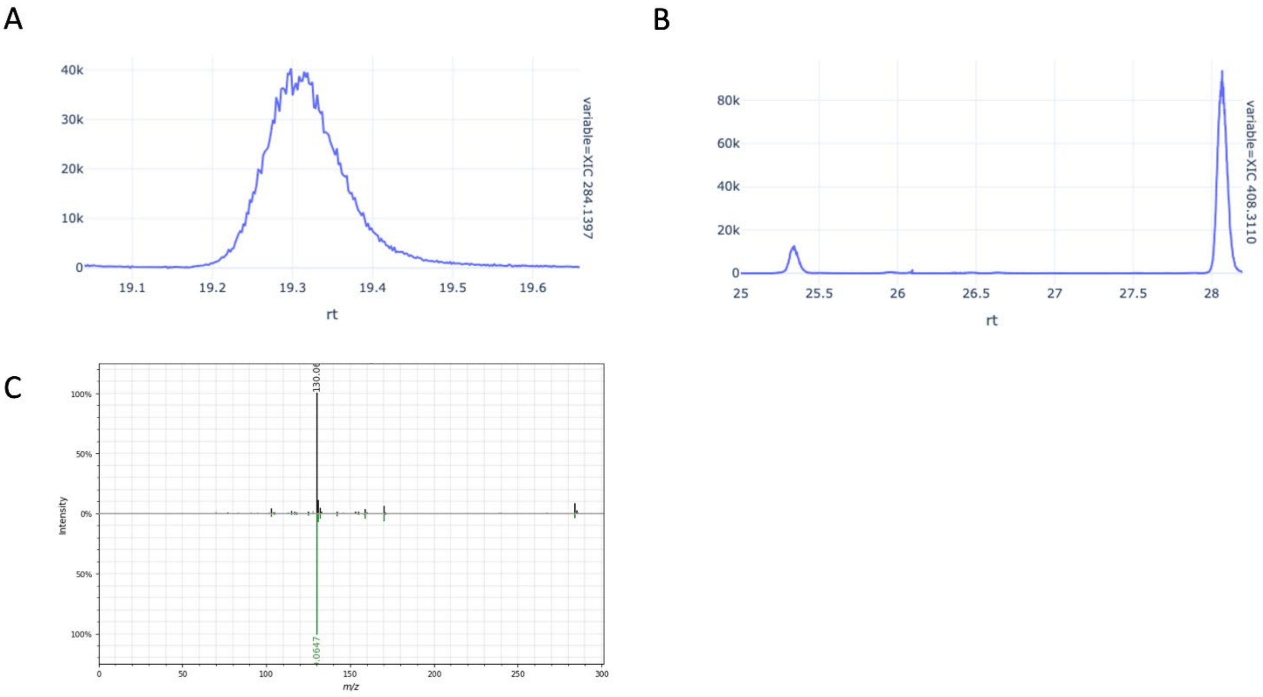

Supplementary Figure 5. Extracted ion chromatograms (XIC) for Brevianamide F (A) and the features annotated as Tricholide A (m/z 408.3110 and RT = 25.32; 28.04) (B) constructed in GNPS Dashboard (Petras et al., 2022). (C) Mirrorplot of MS/MS spectra of Brevianamide F against its reference in GNPS2.
