# Supplementary material for: A multi-omics reciprocal analysis for characterization of bacterial metabolism
Source: Front Mol Biosci. 2025 Mar 20;12:1515276. doi: 10.3389/fmolb.2025.1515276 (PMC11965639; doi:10.3389/fmolb.2025.1515276)
Supplement: Supplementary file 4 [file Table3.docx]

Supplementary Table 3. Results from BLAST between BRA006 Quinolodomicin-A BGC and MiBiG reference BGC0002520 for both sequencing approaches.

A: MiniON

| Query ID | Subject ID | Protein Identity (%) | Alignment Length | Mismatch | Gaps | Query Start | Query End | Subject Start | Subject End | E-value | Bitscore |
| --- | --- | --- | --- | --- | --- | --- | --- | --- | --- | --- | --- |
| ONHDLDEL_08114 | BCK51617.1 | 96.88 | 64 | 2 | 0 | 1 | 64 | 115 | 178 | 8.29E-40 | 129 |
| ONHDLDEL_08115 | BCK51618.1 | 100 | 48 | 0 | 0 | 1 | 48 | 549 | 596 | 1.05E-27 | 95.9 |
| ONHDLDEL_08116 | BCK51618.1 | 97 | 467 | 10 | 1 | 1 | 463 | 1 | 467 | 0 | 915 |
| ONHDLDEL_08117 | BCK51619.1 | 96.77 | 340 | 7 | 1 | 1 | 336 | 87 | 426 | 0 | 644 |
| ONHDLDEL_08118 | BCK51620.1 | 99.39 | 163 | 1 | 0 | 1 | 163 | 24 | 186 | 1.3E-118 | 327 |
| ONHDLDEL_08119 | BCK51621.1 | 89.97 | 319 | 18 | 3 | 1 | 312 | 1 | 312 | 1.4E-153 | 426 |
| ONHDLDEL_08120 | BCK51622.1 | 100 | 72 | 0 | 0 | 1 | 72 | 46 | 117 | 3.52E-44 | 132 |
| ONHDLDEL_08121 | BCK51623.1 | 80.8 | 125 | 23 | 1 | 1 | 124 | 1 | 125 | 2.33E-65 | 191 |
| ONHDLDEL_08122 | BCK51624.1 | 97.78 | 90 | 2 | 0 | 1 | 90 | 37 | 126 | 7.58E-58 | 176 |
| ONHDLDEL_08123 | BCK51624.1 | 97.73 | 44 | 1 | 0 | 1 | 44 | 133 | 176 | 6.55E-25 | 87.8 |
| ONHDLDEL_08124 | BCK51624.1 | 96.25 | 80 | 3 | 0 | 1 | 80 | 237 | 316 | 3.5E-50 | 156 |
| ONHDLDEL_08125 | BCK51624.1 | 88.68 | 53 | 6 | 0 | 2 | 54 | 335 | 387 | 1.07E-30 | 103 |
| ONHDLDEL_08126 | BCK51625.1 | 74.29 | 385 | 79 | 8 | 1 | 374 | 1 | 376 | 5.6E-164 | 457 |
| ONHDLDEL_08127 | BCK51626.1 | 98.31 | 59 | 1 | 0 | 1 | 59 | 1 | 59 | 3.27E-36 | 115 |
| ONHDLDEL_08128 | BCK51626.1 | 97.3 | 74 | 2 | 0 | 1 | 74 | 92 | 165 | 4.71E-47 | 143 |
| ONHDLDEL_08129 | BCK51626.1 | 100 | 64 | 0 | 0 | 1 | 64 | 168 | 231 | 1.92E-40 | 125 |
| ONHDLDEL_08130 | BCK51627.1 | 95 | 40 | 2 | 0 | 12 | 51 | 84 | 123 | 1.54E-21 | 73.9 |
| ONHDLDEL_08132 | BCK51629.1 | 91.84 | 98 | 8 | 0 | 3 | 100 | 821 | 918 | 5.52E-60 | 197 |
| ONHDLDEL_08133 | BCK51628.1 | 95.38 | 519 | 21 | 1 | 1 | 519 | 297 | 812 | 0 | 977 |
| ONHDLDEL_08135 | BCK51629.1 | 98.86 | 88 | 1 | 0 | 28 | 115 | 1097 | 1184 | 3.65E-56 | 181 |
| ONHDLDEL_08136 | BCK51629.1 | 77.32 | 216 | 38 | 2 | 1 | 210 | 842 | 1052 | 1.3E-108 | 332 |
| ONHDLDEL_08137 | BCK51629.1 | 84.54 | 97 | 11 | 1 | 1 | 93 | 710 | 806 | 5.71E-47 | 154 |
| ONHDLDEL_08139 | BCK51629.1 | 98.06 | 412 | 8 | 0 | 1 | 412 | 138 | 549 | 0 | 789 |
| ONHDLDEL_08140 | BCK51629.1 | 98.08 | 52 | 1 | 0 | 1 | 52 | 59 | 110 | 4.44E-28 | 97.8 |
| ONHDLDEL_08141 | BCK51630.1 | 76.18 | 403 | 53 | 7 | 1 | 381 | 1 | 382 | 0 | 551 |
| ONHDLDEL_08142 | BCK51631.1 | 97.25 | 109 | 3 | 0 | 1 | 109 | 204 | 312 | 2.4E-70 | 212 |
| ONHDLDEL_08143 | BCK51631.1 | 96.93 | 163 | 5 | 0 | 1 | 163 | 1 | 163 | 3.4E-112 | 318 |
| ONHDLDEL_08144 | BCK51632.1 | 98.33 | 180 | 3 | 0 | 6 | 185 | 90 | 269 | 3E-123 | 342 |
| ONHDLDEL_08145 | BCK51632.1 | 97.44 | 39 | 1 | 0 | 29 | 67 | 13 | 51 | 7.37E-22 | 78.6 |
| ONHDLDEL_08146 | BCK51633.1 | 83.85 | 650 | 80 | 6 | 1 | 636 | 69 | 707 | 0 | 946 |
| ONHDLDEL_08147 | BCK51633.1 | 90.79 | 76 | 7 | 0 | 1 | 76 | 1312 | 1387 | 1.25E-41 | 138 |
| ONHDLDEL_08151 | BCK51633.1 | 96.17 | 287 | 11 | 0 | 1 | 287 | 1966 | 2252 | 6.5E-174 | 526 |
| ONHDLDEL_08152 | BCK51633.1 | 100 | 195 | 0 | 0 | 1 | 195 | 2459 | 2653 | 2.6E-119 | 370 |
| ONHDLDEL_08153 | BCK51633.1 | 99.43 | 175 | 1 | 0 | 1 | 175 | 2798 | 2972 | 1.4E-112 | 345 |
| ONHDLDEL_08154 | BCK51633.1 | 100 | 219 | 0 | 0 | 1 | 219 | 3009 | 3227 | 1.4E-139 | 426 |
| ONHDLDEL_08156 | BCK51633.1 | 83.27 | 998 | 124 | 12 | 1 | 974 | 3475 | 4453 | 0 | 1459 |
| ONHDLDEL_08157 | BCK51633.1 | 89.47 | 152 | 15 | 1 | 1 | 151 | 4454 | 4605 | 6.46E-70 | 221 |
| ONHDLDEL_08158 | BCK51633.1 | 91.91 | 173 | 13 | 1 | 1 | 173 | 4621 | 4792 | 2.77E-96 | 301 |
| ONHDLDEL_08159 | BCK51633.1 | 98.11 | 53 | 1 | 0 | 1 | 53 | 4909 | 4961 | 4.09E-31 | 105 |
| ONHDLDEL_08161 | BCK51634.1 | 79.38 | 97 | 18 | 2 | 2 | 96 | 99 | 195 | 3.06E-44 | 143 |
| ONHDLDEL_08162 | BCK51634.1 | 96.77 | 93 | 3 | 0 | 12 | 104 | 254 | 346 | 1.87E-60 | 186 |
| ONHDLDEL_08163 | BCK51634.1 | 95.65 | 46 | 2 | 0 | 1 | 46 | 475 | 520 | 1.94E-25 | 89.4 |
| ONHDLDEL_08164 | BCK51635.1 | 93.94 | 33 | 2 | 0 | 1 | 33 | 1 | 33 | 2.17E-17 | 65.9 |
| ONHDLDEL_08166 | BCK51636.1 | 97.92 | 48 | 1 | 0 | 1 | 48 | 163 | 210 | 1.7E-29 | 97.8 |
| ONHDLDEL_08167 | BCK51636.1 | 82.07 | 145 | 20 | 2 | 1 | 142 | 1 | 142 | 1E-79 | 230 |
| ONHDLDEL_08168 | BCK51637.1 | 89.58 | 48 | 4 | 1 | 1 | 47 | 1 | 48 | 1.16E-22 | 82 |
| ONHDLDEL_08169 | BCK51637.1 | 82.3 | 226 | 33 | 5 | 1 | 221 | 49 | 272 | 2.1E-105 | 327 |
| ONHDLDEL_08170 | BCK51637.1 | 92.02 | 401 | 29 | 2 | 1 | 399 | 294 | 693 | 0 | 671 |
| ONHDLDEL_08171 | BCK51637.1 | 88.78 | 196 | 22 | 0 | 1 | 196 | 712 | 907 | 2.4E-113 | 350 |
| ONHDLDEL_08172 | BCK51637.1 | 76.71 | 219 | 30 | 3 | 1 | 208 | 975 | 1183 | 3.49E-94 | 293 |
| ONHDLDEL_08173 | BCK51637.1 | 99.13 | 115 | 1 | 0 | 1 | 115 | 1273 | 1387 | 3.15E-70 | 221 |
| ONHDLDEL_08174 | BCK51637.1 | 97.58 | 124 | 3 | 0 | 1 | 124 | 1391 | 1514 | 1.01E-70 | 223 |
| ONHDLDEL_08175 | BCK51637.1 | 87.18 | 39 | 3 | 1 | 47 | 85 | 1678 | 1714 | 2.79E-17 | 68.6 |
| ONHDLDEL_08176 | BCK51637.1 | 96.55 | 29 | 0 | 1 | 6 | 34 | 1827 | 1854 | 1.19E-13 | 55.1 |
| ONHDLDEL_08179 | BCK51638.1 | 99.04 | 104 | 1 | 0 | 1 | 104 | 211 | 314 | 2.57E-65 | 206 |
| ONHDLDEL_08181 | BCK51638.1 | 99.09 | 220 | 2 | 0 | 1 | 220 | 564 | 783 | 7.9E-139 | 422 |
| ONHDLDEL_08182 | BCK51638.1 | 77.56 | 566 | 68 | 10 | 1 | 537 | 792 | 1327 | 0 | 758 |
| ONHDLDEL_08184 | BCK51638.1 | 86.05 | 86 | 12 | 0 | 29 | 114 | 1849 | 1934 | 1.35E-43 | 146 |
| ONHDLDEL_08187 | BCK51638.1 | 88.66 | 97 | 11 | 0 | 1 | 97 | 5389 | 5485 | 9.85E-52 | 167 |
| ONHDLDEL_08188 | BCK51638.1 | 92.73 | 55 | 4 | 0 | 1 | 55 | 2508 | 2562 | 8.63E-30 | 103 |
| ONHDLDEL_08189 | BCK51638.1 | 97.53 | 81 | 2 | 0 | 1 | 81 | 2578 | 2658 | 7.29E-51 | 164 |
| ONHDLDEL_08190 | BCK51638.1 | 78.95 | 76 | 14 | 2 | 1 | 74 | 2810 | 2885 | 7.41E-33 | 112 |
| ONHDLDEL_08194 | BCK51637.1 | 93.68 | 95 | 6 | 0 | 1 | 95 | 291 | 385 | 2.92E-53 | 171 |
| ONHDLDEL_08197 | BCK51638.1 | 89.35 | 169 | 18 | 0 | 1 | 169 | 5417 | 5585 | 3.43E-92 | 288 |
| ONHDLDEL_08198 | BCK51638.1 | 77.85 | 158 | 32 | 3 | 1 | 156 | 4116 | 4272 | 2.47E-73 | 232 |
| ONHDLDEL_08199 | BCK51638.1 | 98.65 | 74 | 1 | 0 | 1 | 74 | 4287 | 4360 | 7.19E-41 | 134 |
| ONHDLDEL_08200 | BCK51638.1 | 97.35 | 151 | 3 | 1 | 1 | 150 | 4371 | 4521 | 3.19E-83 | 260 |
| ONHDLDEL_08202 | BCK51638.1 | 77.36 | 424 | 78 | 4 | 4 | 418 | 1952 | 2366 | 0 | 590 |
| ONHDLDEL_08205 | BCK51638.1 | 98.44 | 256 | 3 | 1 | 1 | 255 | 5662 | 5917 | 1.8E-151 | 461 |
| ONHDLDEL_08206 | BCK51638.1 | 90.48 | 189 | 17 | 1 | 1 | 188 | 4451 | 4639 | 5.33E-79 | 249 |
| ONHDLDEL_08207 | BCK51638.1 | 95.65 | 46 | 2 | 0 | 48 | 93 | 6241 | 6286 | 4.75E-22 | 83.2 |
| ONHDLDEL_08208 | BCK51638.1 | 100 | 16 | 0 | 0 | 22 | 37 | 6401 | 6416 | 2.99E-07 | 37.4 |
| ONHDLDEL_08209 | BCK51639.1 | 77.44 | 266 | 48 | 4 | 1 | 258 | 1 | 262 | 4.2E-137 | 382 |
| ONHDLDEL_08211 | BCK51640.1 | 91.34 | 127 | 10 | 1 | 1 | 126 | 164 | 290 | 1.62E-69 | 219 |
| ONHDLDEL_08213 | BCK51640.1 | 97.62 | 42 | 1 | 0 | 1 | 42 | 669 | 710 | 8.1E-22 | 79 |
| ONHDLDEL_08214 | BCK51640.1 | 91.11 | 45 | 4 | 0 | 3 | 47 | 798 | 842 | 2.42E-24 | 86.3 |
| ONHDLDEL_08217 | BCK51640.1 | 100 | 56 | 0 | 0 | 1 | 56 | 1291 | 1346 | 2.94E-32 | 109 |
| ONHDLDEL_08218 | BCK51640.1 | 95.35 | 43 | 2 | 0 | 1 | 43 | 1422 | 1464 | 1.91E-23 | 84 |
| ONHDLDEL_08219 | BCK51640.1 | 97.73 | 44 | 1 | 0 | 2 | 45 | 1478 | 1521 | 2.07E-26 | 92 |
| ONHDLDEL_08223 | BCK51640.1 | 79.39 | 456 | 66 | 5 | 1 | 441 | 2343 | 2785 | 0 | 624 |
| ONHDLDEL_08226 | BCK51640.1 | 86.54 | 312 | 32 | 3 | 1 | 306 | 3034 | 3341 | 1.1E-158 | 485 |
| ONHDLDEL_08227 | BCK51640.1 | 96.36 | 55 | 2 | 0 | 1 | 55 | 3493 | 3547 | 2.57E-30 | 103 |
| ONHDLDEL_08228 | BCK51640.1 | 90.4 | 177 | 16 | 1 | 2 | 177 | 3550 | 3726 | 1.17E-97 | 302 |
| ONHDLDEL_08230 | BCK51640.1 | 97.98 | 396 | 8 | 0 | 1 | 396 | 3860 | 4255 | 0 | 749 |
| ONHDLDEL_08232 | BCK51640.1 | 80.15 | 136 | 26 | 1 | 1 | 135 | 4651 | 4786 | 2.49E-57 | 186 |
| ONHDLDEL_08234 | BCK51640.1 | 90.69 | 376 | 23 | 4 | 1 | 370 | 5054 | 5423 | 0 | 620 |
| ONHDLDEL_08235 | BCK51640.1 | 96.61 | 118 | 4 | 0 | 1 | 118 | 5466 | 5583 | 6.84E-66 | 209 |
| ONHDLDEL_08236 | BCK51643.1 | 91.21 | 91 | 7 | 1 | 1 | 90 | 1493 | 1583 | 1.39E-47 | 155 |
| ONHDLDEL_08237 | BCK51640.1 | 92.86 | 28 | 2 | 0 | 1 | 28 | 5717 | 5744 | 7.83E-14 | 55.5 |
| ONHDLDEL_08238 | BCK51640.1 | 100 | 76 | 0 | 0 | 1 | 76 | 6008 | 6083 | 9.57E-49 | 158 |
| ONHDLDEL_08239 | BCK51640.1 | 97.4 | 77 | 2 | 0 | 1 | 77 | 6098 | 6174 | 3.38E-46 | 150 |
| ONHDLDEL_08240 | BCK51641.1 | 83.77 | 154 | 25 | 0 | 1 | 154 | 7091 | 7244 | 2.81E-83 | 260 |
| ONHDLDEL_08243 | BCK51640.1 | 75.1 | 518 | 71 | 10 | 8 | 493 | 6705 | 7196 | 0 | 637 |
| ONHDLDEL_08245 | BCK51641.1 | 97.33 | 75 | 1 | 1 | 7 | 80 | 252 | 326 | 5.94E-44 | 144 |
| ONHDLDEL_08246 | BCK51641.1 | 100 | 53 | 0 | 0 | 1 | 53 | 337 | 389 | 4.01E-31 | 105 |
| ONHDLDEL_08248 | BCK51641.1 | 90.35 | 145 | 13 | 1 | 1 | 144 | 534 | 678 | 2.65E-69 | 220 |
| ONHDLDEL_08249 | BCK51641.1 | 81.45 | 221 | 40 | 1 | 17 | 236 | 859 | 1079 | 8.7E-99 | 309 |
| ONHDLDEL_08251 | BCK51641.1 | 92.63 | 217 | 14 | 2 | 1 | 216 | 1461 | 1676 | 1.6E-128 | 396 |
| ONHDLDEL_08253 | BCK51641.1 | 95.83 | 24 | 1 | 0 | 1 | 24 | 1838 | 1861 | 2.83E-10 | 45.1 |
| ONHDLDEL_08254 | BCK51641.1 | 100 | 36 | 0 | 0 | 1 | 36 | 322 | 357 | 5.98E-19 | 72 |
| ONHDLDEL_08255 | BCK51641.1 | 94.88 | 215 | 11 | 0 | 1 | 215 | 1963 | 2177 | 1.3E-128 | 395 |
| ONHDLDEL_08256 | BCK51640.1 | 83.96 | 106 | 17 | 0 | 1 | 106 | 646 | 751 | 2.18E-53 | 172 |
| ONHDLDEL_08257 | BCK51641.1 | 92.11 | 76 | 6 | 0 | 1 | 76 | 7563 | 7638 | 6.52E-43 | 141 |
| ONHDLDEL_08258 | BCK51641.1 | 82.35 | 153 | 15 | 1 | 1 | 141 | 2438 | 2590 | 9.68E-75 | 235 |
| ONHDLDEL_08260 | BCK51641.1 | 99.28 | 138 | 1 | 0 | 1 | 138 | 3128 | 3265 | 1.2E-84 | 264 |
| ONHDLDEL_08261 | BCK51641.1 | 71.05 | 532 | 97 | 10 | 1 | 503 | 3267 | 3770 | 0 | 650 |
| ONHDLDEL_08262 | BCK51643.1 | 92.65 | 136 | 10 | 0 | 1 | 136 | 583 | 718 | 2.15E-71 | 225 |
| ONHDLDEL_08263 | BCK51641.1 | 87.67 | 73 | 9 | 0 | 1 | 73 | 7511 | 7583 | 1.03E-39 | 131 |
| ONHDLDEL_08264 | BCK51640.1 | 70.37 | 27 | 6 | 1 | 1 | 25 | 6732 | 6758 | 4.78E-06 | 33.9 |
| ONHDLDEL_08265 | BCK51641.1 | 98.57 | 70 | 1 | 0 | 1 | 70 | 4193 | 4262 | 4.07E-42 | 139 |
| ONHDLDEL_08266 | BCK51641.1 | 95.9 | 195 | 7 | 1 | 1 | 194 | 4379 | 4573 | 5.6E-109 | 338 |
| ONHDLDEL_08267 | BCK51641.1 | 100 | 33 | 0 | 0 | 1 | 33 | 4616 | 4648 | 8.11E-16 | 62.4 |
| ONHDLDEL_08268 | BCK51641.1 | 94.66 | 131 | 7 | 0 | 12 | 142 | 4666 | 4796 | 4.34E-70 | 222 |
| ONHDLDEL_08269 | BCK51641.1 | 92.86 | 70 | 5 | 0 | 1 | 70 | 6564 | 6633 | 1.41E-38 | 129 |
| ONHDLDEL_08270 | BCK51641.1 | 98.03 | 152 | 3 | 0 | 1 | 152 | 4996 | 5147 | 2.35E-98 | 306 |
| ONHDLDEL_08272 | BCK51641.1 | 100 | 147 | 0 | 0 | 1 | 147 | 5326 | 5472 | 2.88E-93 | 290 |
| ONHDLDEL_08273 | BCK51641.1 | 96.1 | 77 | 3 | 0 | 1 | 77 | 5494 | 5570 | 1.63E-44 | 146 |
| ONHDLDEL_08274 | BCK51641.1 | 86.61 | 112 | 15 | 0 | 1 | 112 | 5592 | 5703 | 1.7E-58 | 187 |
| ONHDLDEL_08277 | BCK51641.1 | 89.39 | 66 | 5 | 1 | 1 | 64 | 6052 | 6117 | 7.33E-33 | 112 |
| ONHDLDEL_08278 | BCK51641.1 | 79.38 | 97 | 13 | 2 | 1 | 93 | 6123 | 6216 | 3.04E-42 | 139 |
| ONHDLDEL_08279 | BCK51641.1 | 81.7 | 519 | 87 | 3 | 1 | 514 | 6337 | 6852 | 0 | 748 |
| ONHDLDEL_08280 | BCK51641.1 | 96.03 | 302 | 12 | 0 | 1 | 302 | 6917 | 7218 | 0 | 585 |
| ONHDLDEL_08281 | BCK51641.1 | 91.49 | 47 | 3 | 1 | 1 | 46 | 7240 | 7286 | 5.44E-23 | 82.8 |
| ONHDLDEL_08282 | BCK51641.1 | 92.43 | 185 | 14 | 0 | 1 | 185 | 7309 | 7493 | 1.1E-106 | 329 |
| ONHDLDEL_08283 | BCK51641.1 | 89.91 | 228 | 23 | 0 | 1 | 228 | 7494 | 7721 | 6.3E-127 | 392 |
| ONHDLDEL_08287 | BCK51641.1 | 98.88 | 179 | 2 | 0 | 1 | 179 | 8476 | 8654 | 2.3E-113 | 348 |
| ONHDLDEL_08289 | BCK51641.1 | 73.33 | 30 | 6 | 1 | 1 | 28 | 8777 | 8806 | 0.056 | 22.3 |
| ONHDLDEL_08290 | BCK51641.1 | 86.67 | 450 | 52 | 3 | 1 | 445 | 8971 | 9417 | 0 | 728 |
| ONHDLDEL_08291 | BCK51641.1 | 84.54 | 194 | 25 | 1 | 1 | 189 | 9504 | 9697 | 1.22E-88 | 278 |
| ONHDLDEL_08292 | BCK51641.1 | 97.6 | 208 | 5 | 0 | 1 | 208 | 9732 | 9939 | 1.7E-129 | 395 |
| ONHDLDEL_08294 | BCK51642.1 | 87.67 | 219 | 24 | 2 | 1 | 218 | 435 | 651 | 2.7E-106 | 331 |
| ONHDLDEL_08295 | BCK51642.1 | 76.97 | 317 | 56 | 4 | 1 | 309 | 768 | 1075 | 1.1E-131 | 406 |
| ONHDLDEL_08301 | BCK51642.1 | 88.72 | 674 | 56 | 1 | 7 | 680 | 2230 | 2883 | 0 | 963 |
| ONHDLDEL_08302 | BCK51642.1 | 97.13 | 209 | 6 | 0 | 22 | 230 | 2894 | 3102 | 4.1E-129 | 396 |
| ONHDLDEL_08308 | BCK51643.1 | 99.02 | 102 | 1 | 0 | 1 | 102 | 157 | 258 | 8.84E-66 | 210 |
| ONHDLDEL_08309 | BCK51643.1 | 78.18 | 362 | 71 | 6 | 1 | 356 | 394 | 753 | 1.6E-148 | 457 |
| ONHDLDEL_08310 | BCK51643.1 | 84.1 | 239 | 22 | 4 | 1 | 231 | 755 | 985 | 2.8E-123 | 379 |
| ONHDLDEL_08311 | BCK51643.1 | 77.11 | 83 | 19 | 0 | 1 | 83 | 989 | 1071 | 1.31E-32 | 113 |
| ONHDLDEL_08312 | BCK51643.1 | 85.75 | 414 | 36 | 4 | 1 | 392 | 1107 | 1519 | 0 | 620 |
| ONHDLDEL_08313 | BCK51643.1 | 100 | 69 | 0 | 0 | 1 | 69 | 1640 | 1708 | 4.58E-40 | 132 |
| ONHDLDEL_08314 | BCK51643.1 | 95 | 60 | 3 | 0 | 10 | 69 | 1842 | 1901 | 3.36E-37 | 125 |
| ONHDLDEL_08315 | BCK51643.1 | 98.49 | 66 | 1 | 0 | 1 | 66 | 1934 | 1999 | 1.22E-39 | 131 |
| ONHDLDEL_08316 | BCK51643.1 | 96.59 | 88 | 3 | 0 | 1 | 88 | 2013 | 2100 | 1.29E-55 | 177 |
| ONHDLDEL_08317 | BCK51643.1 | 74.3 | 358 | 63 | 6 | 1 | 342 | 2112 | 2456 | 2.1E-120 | 377 |
| ONHDLDEL_08319 | BCK51643.1 | 97.6 | 250 | 5 | 1 | 1 | 249 | 2846 | 3095 | 1.6E-148 | 453 |
| ONHDLDEL_08320 | BCK51643.1 | 98 | 100 | 2 | 0 | 1 | 100 | 3183 | 3282 | 2.45E-61 | 195 |
| ONHDLDEL_08321 | BCK51643.1 | 100 | 122 | 0 | 0 | 1 | 122 | 3348 | 3469 | 1.2E-79 | 249 |
| ONHDLDEL_08322 | BCK51643.1 | 98.8 | 83 | 1 | 0 | 1 | 83 | 3507 | 3589 | 7.23E-50 | 161 |
| ONHDLDEL_08327 | BCK51643.1 | 98.15 | 108 | 2 | 0 | 1 | 108 | 4415 | 4522 | 7.14E-63 | 200 |
| ONHDLDEL_08328 | BCK51643.1 | 98.9 | 91 | 1 | 0 | 1 | 91 | 4525 | 4615 | 1.18E-55 | 178 |
| ONHDLDEL_08329 | BCK51643.1 | 100 | 37 | 0 | 0 | 1 | 37 | 4623 | 4659 | 2.16E-19 | 72.8 |
| ONHDLDEL_08330 | BCK51643.1 | 99.04 | 208 | 2 | 0 | 1 | 208 | 4673 | 4880 | 5.8E-129 | 395 |
| ONHDLDEL_08333 | BCK51644.1 | 85.59 | 111 | 15 | 1 | 1 | 110 | 636 | 746 | 1.38E-50 | 164 |
| ONHDLDEL_08334 | BCK51644.1 | 93.94 | 264 | 7 | 1 | 1 | 264 | 747 | 1001 | 2.8E-157 | 479 |
| ONHDLDEL_08335 | BCK51644.1 | 100 | 74 | 0 | 0 | 1 | 74 | 1228 | 1301 | 5.03E-42 | 138 |
| ONHDLDEL_08336 | BCK51644.1 | 100 | 70 | 0 | 0 | 1 | 70 | 1307 | 1376 | 1.07E-41 | 139 |
| ONHDLDEL_08337 | BCK51644.1 | 98.47 | 131 | 2 | 0 | 1 | 131 | 1575 | 1705 | 7.39E-86 | 266 |
| ONHDLDEL_08338 | BCK51645.1 | 96.45 | 197 | 7 | 0 | 1 | 197 | 3251 | 3447 | 9.6E-121 | 370 |
| ONHDLDEL_08339 | BCK51644.1 | 94.95 | 99 | 5 | 0 | 1 | 99 | 1939 | 2037 | 1.02E-59 | 189 |
| ONHDLDEL_08340 | BCK51644.1 | 72.73 | 44 | 9 | 1 | 1 | 41 | 2116 | 2159 | 6.13E-15 | 59.3 |
| ONHDLDEL_08341 | BCK51645.1 | 97.26 | 73 | 2 | 0 | 1 | 73 | 3766 | 3838 | 3.42E-41 | 135 |
| ONHDLDEL_08342 | BCK51646.1 | 100 | 35 | 0 | 0 | 1 | 35 | 2576 | 2610 | 2.85E-21 | 77 |
| ONHDLDEL_08344 | BCK51644.1 | 92.71 | 713 | 26 | 4 | 1 | 699 | 2833 | 3533 | 0 | 1157 |
| ONHDLDEL_08345 | BCK51644.1 | 98.66 | 149 | 2 | 0 | 1 | 149 | 3580 | 3728 | 1.6E-92 | 286 |
| ONHDLDEL_08347 | BCK51645.1 | 85.28 | 428 | 46 | 6 | 1 | 418 | 691 | 1111 | 0 | 645 |
| ONHDLDEL_08348 | BCK51645.1 | 77.22 | 338 | 47 | 4 | 1 | 322 | 1240 | 1563 | 7.7E-144 | 442 |
| ONHDLDEL_08349 | BCK51645.1 | 97.5 | 80 | 2 | 0 | 1 | 80 | 1578 | 1657 | 5.23E-52 | 167 |
| ONHDLDEL_08350 | BCK51645.1 | 97.4 | 77 | 2 | 0 | 1 | 77 | 1661 | 1737 | 4.16E-46 | 150 |
| ONHDLDEL_08351 | BCK51645.1 | 97.5 | 80 | 2 | 0 | 1 | 80 | 1745 | 1824 | 1.39E-48 | 157 |
| ONHDLDEL_08352 | BCK51645.1 | 98.36 | 61 | 1 | 0 | 1 | 61 | 1829 | 1889 | 2.04E-36 | 121 |
| ONHDLDEL_08354 | BCK51645.1 | 97.41 | 116 | 3 | 0 | 1 | 116 | 2186 | 2301 | 2.16E-69 | 220 |
| ONHDLDEL_08356 | BCK51645.1 | 98.34 | 362 | 6 | 0 | 12 | 373 | 2513 | 2874 | 0 | 688 |
| ONHDLDEL_08357 | BCK51645.1 | 93.4 | 318 | 20 | 1 | 10 | 326 | 3096 | 3413 | 0 | 561 |
| ONHDLDEL_08359 | BCK51646.1 | 95.71 | 140 | 5 | 1 | 10 | 149 | 2433 | 2571 | 8.71E-80 | 251 |
| ONHDLDEL_08360 | BCK51645.1 | 94.29 | 175 | 10 | 0 | 1 | 175 | 3892 | 4066 | 3.8E-106 | 327 |
| ONHDLDEL_08361 | BCK51645.1 | 94.74 | 133 | 7 | 0 | 4 | 136 | 4179 | 4311 | 4.1E-71 | 224 |
| ONHDLDEL_08362 | BCK51645.1 | 77.1 | 393 | 71 | 5 | 1 | 383 | 4327 | 4710 | 1.8E-166 | 509 |
| ONHDLDEL_08363 | BCK51646.1 | 99.59 | 246 | 1 | 0 | 1 | 246 | 120 | 365 | 5.7E-161 | 488 |
| ONHDLDEL_08365 | BCK51645.1 | 88.1 | 294 | 35 | 0 | 1 | 294 | 581 | 874 | 9.5E-160 | 490 |
| ONHDLDEL_08366 | BCK51646.1 | 77.73 | 422 | 67 | 5 | 48 | 454 | 1012 | 1421 | 2.2E-168 | 519 |
| ONHDLDEL_08368 | BCK51646.1 | 100 | 26 | 0 | 0 | 1 | 26 | 1619 | 1644 | 1.71E-13 | 55.5 |
| ONHDLDEL_08369 | BCK51646.1 | 74.41 | 211 | 29 | 5 | 1 | 199 | 1669 | 1866 | 1.51E-79 | 255 |
| ONHDLDEL_08371 | BCK51647.1 | 88.11 | 185 | 22 | 0 | 1 | 185 | 545 | 729 | 9.55E-94 | 291 |
| ONHDLDEL_08372 | BCK51645.1 | 94.29 | 105 | 6 | 0 | 1 | 105 | 3786 | 3890 | 1.7E-62 | 199 |
| ONHDLDEL_08375 | BCK51646.1 | 98 | 399 | 8 | 0 | 1 | 399 | 2956 | 3354 | 0 | 743 |
| ONHDLDEL_08376 | BCK51646.1 | 98.45 | 258 | 4 | 0 | 1 | 258 | 3390 | 3647 | 1.4E-163 | 495 |
| ONHDLDEL_08377 | BCK51647.1 | 99.45 | 183 | 1 | 0 | 1 | 183 | 40 | 222 | 9.1E-123 | 376 |
| ONHDLDEL_08378 | BCK51647.1 | 98.72 | 156 | 2 | 0 | 1 | 156 | 274 | 429 | 2.2E-99 | 309 |
| ONHDLDEL_08380 | BCK51647.1 | 77.75 | 346 | 69 | 3 | 1 | 341 | 668 | 1010 | 9.6E-168 | 505 |
| ONHDLDEL_08381 | BCK51647.1 | 94.67 | 150 | 3 | 1 | 1 | 150 | 1264 | 1408 | 3.83E-89 | 277 |
| ONHDLDEL_08383 | BCK51647.1 | 99.41 | 168 | 1 | 0 | 1 | 168 | 1497 | 1664 | 6.7E-106 | 325 |
| ONHDLDEL_08384 | BCK51648.1 | 93.08 | 159 | 11 | 0 | 1 | 159 | 1 | 159 | 5.88E-98 | 302 |
| ONHDLDEL_08385 | BCK51648.1 | 97.52 | 121 | 3 | 0 | 1 | 121 | 294 | 414 | 1.75E-71 | 227 |
| ONHDLDEL_08386 | BCK51648.1 | 98.25 | 228 | 4 | 0 | 1 | 228 | 697 | 924 | 1.8E-145 | 442 |
| ONHDLDEL_08387 | BCK51648.1 | 93.61 | 501 | 23 | 5 | 1 | 494 | 953 | 1451 | 0 | 820 |
| ONHDLDEL_08388 | BCK51648.1 | 97.67 | 86 | 2 | 0 | 1 | 86 | 1461 | 1546 | 1.8E-51 | 166 |
| ONHDLDEL_08389 | BCK51648.1 | 98.86 | 88 | 1 | 0 | 1 | 88 | 1557 | 1644 | 2.57E-57 | 184 |
| ONHDLDEL_08391 | BCK51648.1 | 98.83 | 257 | 3 | 0 | 1 | 257 | 1909 | 2165 | 2.2E-162 | 494 |
| ONHDLDEL_08393 | BCK51648.1 | 98.63 | 146 | 2 | 0 | 1 | 146 | 2570 | 2715 | 7.21E-91 | 281 |
| ONHDLDEL_08394 | BCK51648.1 | 100 | 33 | 0 | 0 | 1 | 33 | 2725 | 2757 | 7.6E-17 | 64.7 |
| ONHDLDEL_08395 | BCK51648.1 | 97.66 | 128 | 3 | 0 | 1 | 128 | 2807 | 2934 | 5.5E-77 | 251 |
| ONHDLDEL_08396 | BCK51649.1 | 97.2 | 357 | 10 | 0 | 1 | 357 | 123 | 479 | 0 | 680 |
| ONHDLDEL_08397 | BCK51649.1 | 96.55 | 87 | 3 | 0 | 1 | 87 | 516 | 602 | 2.17E-50 | 162 |
| ONHDLDEL_08398 | BCK51649.1 | 100 | 123 | 0 | 0 | 1 | 123 | 675 | 797 | 1.65E-75 | 238 |
| ONHDLDEL_08401 | BCK51649.1 | 94.92 | 118 | 5 | 1 | 18 | 135 | 1369 | 1485 | 2.45E-64 | 205 |
| ONHDLDEL_08402 | BCK51649.1 | 99.09 | 219 | 2 | 0 | 1 | 219 | 1552 | 1770 | 7.8E-144 | 446 |
| ONHDLDEL_08403 | BCK51650.1 | 97.58 | 124 | 3 | 0 | 1 | 124 | 176 | 299 | 1.61E-82 | 240 |
| ONHDLDEL_08404 | BCK51650.1 | 100 | 36 | 0 | 0 | 1 | 36 | 315 | 350 | 8.4E-22 | 77.8 |
| ONHDLDEL_08405 | BCK51651.1 | 95.58 | 113 | 5 | 0 | 2 | 114 | 2 | 114 | 3.25E-73 | 212 |
| ONHDLDEL_08406 | BCK51651.1 | 95.65 | 46 | 2 | 0 | 1 | 46 | 122 | 167 | 4.3E-23 | 81.3 |
| ONHDLDEL_08407 | BCK51651.1 | 88.42 | 95 | 9 | 2 | 1 | 94 | 166 | 259 | 2.38E-54 | 163 |
| ONHDLDEL_08408 | BCK51652.1 | 94.26 | 296 | 12 | 2 | 1 | 293 | 646 | 939 | 0 | 539 |
| ONHDLDEL_08410 | BCK51652.1 | 93.33 | 30 | 2 | 0 | 1 | 30 | 538 | 567 | 2.18E-14 | 59.3 |
| ONHDLDEL_08411 | BCK51652.1 | 97.88 | 425 | 9 | 0 | 1 | 425 | 101 | 525 | 0 | 817 |
| ONHDLDEL_08412 | BCK51653.1 | 99.05 | 105 | 1 | 0 | 1 | 105 | 676 | 780 | 2.82E-65 | 209 |
| ONHDLDEL_08413 | BCK51653.1 | 98.53 | 204 | 3 | 0 | 9 | 212 | 425 | 628 | 3.7E-136 | 399 |
| ONHDLDEL_08414 | BCK51653.1 | 94.74 | 114 | 6 | 0 | 21 | 134 | 131 | 244 | 1.27E-67 | 213 |
| ONHDLDEL_08415 | BCK51653.1 | 97.14 | 70 | 2 | 0 | 1 | 70 | 2 | 71 | 4.32E-39 | 130 |
| ONHDLDEL_08418 | BCK51656.1 | 84.56 | 149 | 2 | 2 | 1 | 148 | 1 | 129 | 1.43E-81 | 234 |
| ONHDLDEL_08419 | BCK51657.1 | 75 | 28 | 7 | 0 | 13 | 40 | 1 | 28 | 5.49E-09 | 42.4 |

B: Illumina

| Query ID | Subject ID | Protein Identity (%) | Alignment Length | Mismatch | Gaps | Query Start | Query End | Subject Start | Subject End | E-value | Bitscore |
| --- | --- | --- | --- | --- | --- | --- | --- | --- | --- | --- | --- |
| OPPBIMDH_03602 | BCK51645.1 | 96.725 | 794 | 26 | 0 | 28 | 821 | 3917 | 4710 | 0 | 1472 |
| OPPBIMDH_03603 | BCK51646.1 | 94.572 | 958 | 51 | 1 | 1 | 958 | 1 | 957 | 0 | 1673 |
| OPPBIMDH_03604 | BCK51646.1 | 97.845 | 2645 | 55 | 1 | 1 | 2645 | 1005 | 3647 | 0 | 5048 |
| OPPBIMDH_03605 | BCK51647.1 | 97.596 | 1664 | 40 | 0 | 1 | 1664 | 1 | 1664 | 0 | 3213 |
| OPPBIMDH_03606 | BCK51648.1 | 98.736 | 3165 | 39 | 1 | 1 | 3164 | 1 | 3165 | 0 | 6080 |
| OPPBIMDH_03607 | BCK51649.1 | 96.99 | 1827 | 47 | 2 | 1 | 1827 | 1 | 1819 | 0 | 3424 |
| OPPBIMDH_03608 | BCK51650.1 | 99.143 | 350 | 3 | 0 | 1 | 350 | 1 | 350 | 0 | 696 |
| OPPBIMDH_03609 | BCK51651.1 | 99.612 | 258 | 1 | 0 | 22 | 279 | 2 | 259 | 0 | 505 |
| OPPBIMDH_03610 | BCK51652.1 | 98.085 | 940 | 18 | 0 | 1 | 940 | 1 | 940 | 0 | 1805 |
| OPPBIMDH_03611 | BCK51653.1 | 98.002 | 951 | 19 | 0 | 1 | 951 | 2 | 952 | 0 | 1820 |
| OPPBIMDH_03612 | BCK51655.1 | 97.817 | 458 | 9 | 1 | 1 | 458 | 1 | 457 | 0 | 852 |
| OPPBIMDH_03613 | BCK51656.1 | 86.667 | 165 | 2 | 1 | 1 | 165 | 1 | 145 | 1.36E-97 | 272 |
| OPPBIMDH_03614 | BCK51657.1 | 97.38 | 229 | 6 | 0 | 1 | 229 | 1 | 229 | 6.02E-161 | 438 |
| OPPBIMDH_03615 | BCK51658.1 | 98.507 | 469 | 7 | 0 | 1 | 469 | 1 | 469 | 0 | 885 |
| OPPBIMDH_03616 | BCK51659.1 | 97.285 | 221 | 6 | 0 | 1 | 221 | 1 | 221 | 1.73E-149 | 408 |
| OPPBIMDH_03617 | BCK51660.1 | 100 | 313 | 0 | 0 | 1 | 313 | 1 | 313 | 0 | 622 |
| OPPBIMDH_03618 | BCK51661.1 | 99.184 | 245 | 2 | 0 | 1 | 245 | 1 | 245 | 3.41E-169 | 460 |
| OPPBIMDH_03619 | BCK51662.1 | 96.25 | 80 | 3 | 0 | 1 | 80 | 1 | 80 | 3.93E-54 | 156 |
| OPPBIMDH_03620 | BCK51663.1 | 97.619 | 126 | 3 | 0 | 1 | 126 | 1 | 126 | 1.51E-89 | 249 |
